# Solubility Behavior of Salicylic Acid in Aqueous Alcohol Mixtures: Interplay of Temperature, Solvent Composition, and Thermodynamic Modeling

**DOI:** 10.1021/acsomega.6c04286

**Published:** 2026-07-24

**Authors:** Alessandro C. Galvão, Guilherme A. Mariane, Raquel Bordignon, Igor G. Kaiser, Pedro F. Arce, Weber S. Robazza

**Affiliations:** † Laboratory ApTher—Applied Thermophysics, Department of Food and Chemical Engineering, Santa Catarina State University—UDESC, 89870-000 Pinhalzinho, Santa Catarina, Brazil; ‡ Laboratory of Applied Thermodynamics and Separation Processes, Department of Chemical Engineering, Engineering School of Lorena, University of São Paulo—USP, 12602-810 Lorena, São Paulo, Brazil

## Abstract

Salicylic acid is widely used in pharmaceutical and cosmetic applications, where reliable solubility data are critical for optimizing crystallization-based purification processes. While literature reports values for neat solvents, comprehensive measurements in binary water–alcohol mixtures over the full composition range remain scarce. In this work, the mole fraction solubility of salicylic acid was experimentally determined in the binary mixtures of water + methanol, water + ethanol, and water +2-propanol over the entire solvent composition range at temperatures from 283.2 to 323.2 K at atmospheric pressure using a gravimetric method. The experimental data were analyzed using analytical solutions of groups (ASOG), PC-SAFT, and PSRK thermodynamic models to describe the solid–liquid equilibrium behavior. PC-SAFT showed the best overall performance, yielding the lowest deviations, followed by PSRK and ASOG. The solubility increases monotonically with temperature in all systems, indicating an endothermic dissolution process. In mixed solvents, the solubility decreases sharply with an increasing water mole fraction, supporting the antisolvent effect of water. This behavior is associated with solvent polarity and specific solute–solvent interactions. Alcohols act as effective cosolvents, increasing solubility as the solvent polarity decreases. The new data sets fill important gaps in the literature and provide a consistent basis for thermodynamic modeling, supporting the improved design of crystallization and separation processes involving salicylic acid.

## Graphical abstract

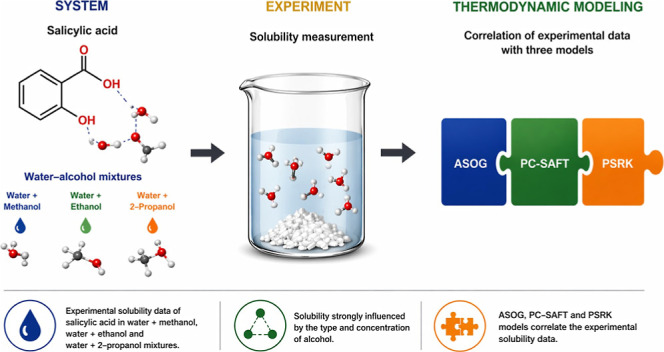

## 1 Introduction

Salicylic acid, an *ortho*-hydroxybenzoic acid derived from benzoic acid, is used in multiple industrial sectors due to its versatile applications. It is a key precursor in the synthesis of acetylsalicylic acid, a widely consumed analgesic and anti-inflammatory drug.^1^ In dermatology, its keratolytic and antibacterial properties make it effective for acne treatment and skin exfoliation.^2^ Derivatives such as methyl salicylate are also used in the production of dyes and fragrances, highlighting their importance in pharmaceutical, cosmetic, and chemical industries.^3^

Crystallization of salicylic acid is a key purification step, in which solubility directly influences crystal yield and purity.^4,5^ The solvent chosen for this process greatly affects its success, as it needs to balance solubility at different temperatures to ensure effective crystal formation and high purity.^6^ Selecting a solvent with the right solubility characteristics allows for better yield and well-formed crystals, meeting the needs of chemical processes.^7^

In industrial crystallization processes, solvent selection remains a major challenge because inadequate solvent systems may lead to a low crystal yield, reduced product purity, and limited control over crystal properties. Pure solvents often fail to provide the balance required between high solubility at elevated temperatures and efficient crystallization during cooling or antisolvent addition. Consequently, mixed solvent systems have gained increasing attention as a strategy to improve the efficiency and overall process performance in pharmaceutical and fine chemical applications.

In addition to experimental measurements, thermodynamic modeling provides insights into the solid–liquid equilibrium behavior as a function of temperature, solvent composition, and molecular interactions.^8^ Predictive tools such as UNIFAC and analytical solutions of groups (ASOG), based on group contribution theory, can be applied to mixtures of nonpolar and polar molecules.^9^ Despite limitations in capturing specific molecular interactions, such as strong hydrogen bonding,^10^ these models can support industrial processes like drug formulation and crystallization by improving solubility predictions and process optimization.^11,12^

A review of the literature, summarized in Table 1 (34 neat solvents) and Table 2 (39 binary solvent mixtures), shows that gravimetric, spectrophotometric, and high-performance liquid chromatography methods are frequently applied, covering a temperature range of 243–348 K. Collectively, these studies provide valuable thermodynamic insights into the dissolution behavior of salicylic acid in a wide range of solvent media.

**Table 1:** Research on the Solubility of Salicylic Acid in Neat Solvents^a^

solvent	*T* (K)	method	modeling	refs
(1), (2), (16), (17), (20), (21)	283–323	gravimetric	---	^5^
(1), (3)	293–323	HPLC	Jouyban–Acree	^13^
(1), (3), (21), (26), (27)	298–348	gravimetric	---	^4^
(2), (16), (17), (21)	243–283	gravimetric	---	^14^
(3), (4), (6), (10), (11), (12)	278–318	gravimetric	Wilson, NRTL, UNIQUAC	^15^
(1), (3), (21)	293–313	spectrophotometry	---	^16^
(4), (5), (6), (7), (8), (9), (10), (13), (14), (15), (17), (18), (19), (21), (22), (25)	298	spectrophotometry	---	^17^
(1), (4), (5), (17)	298–338	spectrophotometry	Jouyban–Acree	^18^
(3), (23), (24)	298–328	spectrophotometry	Jouyban–Acree	^19^
(1), (2), (3), (21), (25), (28)	298	HPLC	Wilson, NRTL	^20^
(1)	288–323	spectrophotometry	CPA	^21^
(1)	302–313	titration	---	^22^
(1), (25)	283–313	spectrophotometry	---	^23^
(1), (2), (3), (4), (6), (17), (21), (23), (25), (29), (30), (31), (32), (33), (34)	304	titration	---	^24^

a (1) water, (2) methanol, (3) ethanol, (4) 1-propanol, (5) 2-propanol, (6) 1-butanol, (7) 2-butanol, (8) 2-methyl-1-propanol, (9) 2-methyl-2-propanol, (10) 1-pentanol, (11) 1-hexanol, (12) 1-heptanol, (13) 1-octanol, (14) dibutyl ether, (15) tetrahydrofuran, (16) acetonitrile, (17) acetone, (18) 2-butanone, (19) cyclohexanone, (20) acetic acid, (21) ethyl acetate, (22) butyl acetate, (23) propylene glycol, (24) *N*-methyl-2-pyrrolidone, (25) 1,4-dioxane, (26) carbon tetrachloride, (27) xylene, (28) polyethylene glycol, (29) benzene, (30) chloroform, (31) cyclohexanol, (32) benzyl alcohol, (33) ethylene glycol, and (34) glycerin.

**Table 2:** Research on the Solubility of Salicylic Acid in Binary Solvent Mixtures^a^

solvent	*T* (K)	method	modeling	refs
(4)	293–323	HPLC	Jouyban–Acree	^13^
(4), (13)	293–313	spectrophotometry	---	^16^
(6), (7), (8)	298	spectrophotometry	Jouyban–Acree	^18^
(11), (12), (35)	298	spectrophotometry	Jouyban–Acree	^19^
(2), (4), (5), (9), (13)	298	HPLC	Wilson, NRTL	^20^
(9)	283–313	spectrophotometry	---	^23^
(6), (9), (13), (14), (15), (16), (17), (18), (19), (20), (21), (22), (23), (24), (25), (26), (27), (28), (29), (30), (31), (32), (33), (36), (37), (38), (39)	304	titration	---	^24^

a (1) water + ethylene glycol, (2) water + polyethylene glycol, (3) water + propylene glycol, (4) water + ethanol, (5) water + methanol, (6) water +1-propanol, (7) water +2-propanol, (8) water + acetone, (9) water +1,4-dioxane, (10) water +1-butanol, (11) ethanol + propylene glycol, (12) ethanol + *N*-methyl-2-pyrrolidone, (13) ethanol + ethyl acetate, (14) ethanol +1,4-dioxane, (15) ethanol + benzene, (16) ethanol + chloroform, (17) methanol + ethyl acetate, (18) methanol +1,4-dioxane, (19) methanol + benzene, (20) methanol + chloroform, (21) 1-propanol + ethyl acetate, (22) 1-propanol +1,4-dioxane, (23) 1-propanol + benzene, (24) 1-propanol + chloroform, (25) 1-butanol + ethyl acetate, (26) 1-butanol +1,4-dioxane, (27) 1-butanol + benzene, (28) 1-butanol + chloroform, (29) acetone + water, (30) acetone +1,4-dioxane, (31) acetone + ethyl acetate, (32) acetone + benzene, (33) acetone + chloroform, (34) propylene glycol + ethanol, (35) propylene glycol + *N*-methyl-2-pyrrolidone, (36) propylene glycol +1,4-dioxane, (37) propylene glycol + ethyl acetate, (38) propylene glycol + chloroform, (39) ethylene glycol +1,4-dioxane.

However, several limitations hinder their applicability for a rigorous thermodynamic analysis and process design. Many studies report solubility in mass-based units (e.g., g/L) without providing density data, which prevents reliable conversion to mole fractions, which is a key requirement for thermodynamic modeling. Only six publications apply computational models, and none employ predictive group contribution approaches such as UNIFAC or ASOG. Furthermore, limited composition ranges in complex binary mixtures hinder a comprehensive understanding of solvent effects that are relevant to crystallization processes.

The critical temperature, critical pressure, and acentric factors of the pure components employed in the PSRK equation of state are presented in Table 8.

**Table 3:** Source and Purity of the Chemicals Used in This Work

component	source	CAS No	mass fraction^a^	purification
methanol	Sigma-Aldrich (France)	67–56–1	≥0.999	-
ethanol	Sigma-Aldrich (Canada)	64–17–5	≥0.999	-
2-propanol	Sigma-Aldrich (France)	67–63–0	≥0.999	-
salicylic acid	Sigma-Aldrich (China)	69–72–7	≥0.990	drying

a The purity of the chemical reagents is provided by the suppliers.

**Table 4:** Solubility of Salicylic Acid (*x*_SA_^Exp^) in Pure Solvents at 95.2 kPa ± 0.3 kPa Compared to Literature Data (*x*_SA_^Lit^)­^a^

*T* (K)	***x***_SA_^Exp^	***x***_SA_^Lit^
Water		
283.2	0.00015	0.00016 ^5^
293.2	0.00021	0.00021 ^5^; 0.00023 ^13^
303.2	0.00032	0.00030 ^5^; 0.00038 ^4^; 0.00035 ^13^
313.2	0.00047	0.00045 ^5^; 0.00054 ^4^; 0.00051 ^13^
323.2	0.00070	0.00068 ^5^; 0.00082 ^4^; 0.00076 ^13^
Methanol		
283.2	0.09918	0.09935 ^5^; 0.10000 ^14^
293.2	0.11712	0.11772 ^5^
303.2	0.13795	0.13937 ^5^
313.2	0.16232	0.16359 ^5^
323.2	0.18976	0.19168 ^5^
Ethanol		
283.2	0.11628	0.11690 ^15^
293.2	0.13473	0.13580 ^15^; 0.13100 ^13^; 0.13480 ^16^
303.2	0.15621	0.15623 ^4^; 0.15660 ^15^; 0.15500 ^13^; 0.14050 ^16^
313.2	0.18119	0.18536 ^4^; 0.18210 ^15^; 0.18400 ^13^; 0.16050 ^16^
323.2	0.20850	0.21364 ^4^; 0.22000 ^13^

a Standard uncertainties *u* are *u*(*T*) = 0.1 K and *u*_r_(*x*_SA_) = 0.02.

**Table 5:** ASOG Interaction Parameters^a^

*k*	l	*m*_k,l_	*m*_l,k_	*n*_k,l_	*n*_l,k_
CH_2_	ArCH	–0.7457	146.0	0.7297	–176.8
CH_2_	H_2_O	–0.2727	–277.3	0.5045	–2382.3
CH_2_	OH	–41.2503	7686.4	4.7125	–3060.0
CH_2_	COOH	9.7236	–3797.5	–10.9719	4022.0
ArCH*	H_2_O*	2.8074	–1057.3	–0.4918	–1008.8
ArCH	OH	2.2682	–1111.5	–0.5859	–939.1
ArCH	COOH	1.4405	–492.9	–0.2256	–213.7
H_2_O	OH	1.4318	–280.2	–5.8341	1582.5
H_2_O	COOH	–0.4492	7.4	–2.1113	779.7

a Interaction parameters marked with an asterisk were estimated in this work by using experimental phase equilibrium (solubility) data. All remaining parameters were taken from the literature.^29^

**Table 6:** PC-SAFT Pure Component Parameters, Along With Deviations in Vapor Pressure and Liquid Density^a^

component	*m*	σ × 10^10^ (m)	ε/*k* (K)	$$k^{A_{i}B_{i}}$$	$\varepsilon^{A_{i}B_{i}}/k$ (K)	Δ*P* ^sat^	Δρ
methanol	1.3552	3.4085	195.74	0.0324	2891.41	0.48	0.69
ethanol	2.4708	3.3704	202.41	0.0306	2631.15	0.41	0.71
2-propanol	2.9541	3.1967	210.62	0.0255	2182.45	0.39	0.66
water	1.1845	2.9748	375.51	0.0353	2516.78	0.42	0.68
salicylic acid	1.8146	4.5181	626.75	0.0351	1926.03	0.35	0.56

a All parameters reported in this table were obtained by regression to vapor pressure and saturated liquid density data (DIPPR database);^41^ $\Delta P^{sat}=\frac{\left|P^{sat,\exp}-P^{sat,calc}\right|}{P^{sat,\exp}}\times 100$ ; $\Delta \rho =\frac{\left|\rho^{\exp}-\rho^{calc}\right|}{\rho^{\exp}}\times 100$ .

**Table 7:** Binary Interaction Parameters of the PC-SAFT Model^a^

***k***_*ij*_			
(1)(2)	(1)(3)	(1)(4)	(1)(5)
0.1524–112.2074/*T*	0.2974–126.1418/*T*	0.1146–133.0418/*T*	0.0956–121.5214/*T*
(2)(1)	(2)(3)	(2)(4)	(2)(5)
0.2718–119.3512/*T*	0.1542–111.7400/*T*	0.3124–121.2108/*T*	0.2654–85.6325/*T*
(3)(1)	(3)(2)	(3)(4)	(3)(5)
0.2871–131.6207/*T*	0.2028–107.3600/*T*	0.0000	0.0000
(4)(1)	(4)(2)	(4)(3)	(4)(5)
0.3041–123.0741/*T*	0.1806–132.4107/*T*	0.0000	0.0000
(5)(1)	(5)(2)	(5)(3)	(5)(4)
0.1280–25.5214/*T*	0.1060–15.4108/*T*	0.0000	0.0000

a (1) salicylic acid, (2) water, (3) methanol, (4) ethanol, and (5) 2-propanol, *k*_*ii*_ = 0; all binary interaction parameters were regressed using experimental phase equilibrium (solubility) data within this work.

**Table 8:** Critical Properties of Components Used in This Work

component	*T*_C_ (K)	*P*_C_ (MPa)	ω
methanol^41^	512.50	8.084	0.5658
ethanol^41^	514.02	6.137	0.6436
2-propanol^41^	508.30	4.764	0.6669
water^41^	647.13	22.055	0.3449
salicylic acid	739.02^39^	5.17^39^	0.8327^40^

In binary mixtures of water and alcohols, salicylic acid exhibits very low solubility in pure water, whereas alcohols, such as methanol, ethanol, and 2-propanol, substantially enhance its dissolution. The solubility decreases sharply with increasing water content, indicating that water acts as an antisolvent in these systems, whereas the alcohols function as effective cosolvents. This dependence enables controlled crystallization through the modulation of the medium, particularly by increasing the water mole fraction. However, comprehensive data covering the entire composition range have remained limited.

Therefore, this study aims to provide systematic solubility data of salicylic acid in the binary mixtures of water with methanol, ethanol, and 2-propanol over the complete mole fraction range and at temperatures from 283.2 to 323.2 K. Solubility was determined gravimetrically and expressed in mole fraction units. Furthermore, the PSRK and PC-SAFT equations of state (EoS), together with the ASOG excess Gibbs energy model, were applied to correlate and analyze the experimental data. This approach enables a deeper understanding of solute–solvent interactions and supports the development of efficient crystallization and separation processes.

## 2 Experimental Study

The solubility of salicylic acid in water–methanol, water–ethanol, and water-2-propanol mixtures was measured using a gravimetric technique, following approaches described in earlier studies.^25,26^ The alcohols were used as received, without further purification. Distilled water was deionized to a conductivity of 0.054 μS·cm^–1^. Before the experiments, salicylic acid was dried in an oven under hot air circulation at 353.2 K for at least 6 h. Table 3 lists the sources and purity of the chemicals used.

The experiments to determine solid–liquid equilibrium were carried out in a 100 mL jacketed glass vessel connected to a thermostatic bath, which maintained temperature within ±0.1 K. The water–alcohol mixtures were prepared in advance using an analytical balance (±0.001 g). The repeatability of mass measurements was verified by replicate weighing, resulting in a relative standard uncertainty below 0.05%. The solutions were stored at a controlled temperature (≈277.2 K) until use. This uncertainty was later propagated into the final mole fraction calculations.

Known amounts of salicylic acid and solvent mixture were placed in an equilibrium vessel and stirred for 3 h to allow mass transfer, followed by 5 h of settling to ensure phase separation. Thereafter, three samples were taken from the liquid phase under sealed conditions and dried in a hot air oven at 353.2 K until constant mass was reached, with measurements performed every 8 h.

This temperature is well below the melting point of salicylic acid (approximately 432 K), thereby minimizing the possibility of thermal degradation during drying. According to the literature,^27^ salicylic acid may exhibit sublimation only at temperatures higher than the drying temperature employed in this work. Therefore, the chosen conditions were considered sufficiently low to avoid significant sublimation or thermal decomposition during solvent evaporation.

The equilibrium cell was hermetically sealed throughout the experiments, including during sampling, and contained the liquid phase with a negligible headspace in order to minimize preferential solvent evaporation. Under the experimental conditions employed in this work, significant changes in the solvent composition during the measurements were considered unlikely.

No solid-phase characterization techniques, such as XRD or DSC, were performed. However, salicylic acid is known^5^ to exhibit a stable polymorphic form under ambient conditions, and no polymorphic transitions or solvate formation have been reported for similar aqueous–alcoholic systems under comparable experimental conditions. Therefore, the solid phase was assumed to remain unchanged throughout the experiments.

The mole fraction of the ternary liquid phase in equilibrium with the solid was determined by mass balance using the salicylic acid content in the sample, the original solvent composition, and the molecular weights of all of the components. The solubility was calculated as the arithmetic mean of three independent measurements. The standard deviation of these replicates was used to assess experimental repeatability.

The uncertainty associated with the mole fraction solubility (*x*) was evaluated by propagating the uncertainties from the experimental measurements, including the mass of solute and solvent, as shown in eq 1, following standard uncertainty propagation procedures reported in the literature.^28^ The sources of uncertainty considered in this work include the balance accuracy, temperature stability, and the repeatability of the gravimetric procedure.

1 $$u\left(x\right)=\sqrt{\sum_{i}{\left(\frac{\partial x}{\partial y_{i}}u\left(y_{i}\right)\right)}^{2}}$$

In eq 1, *y*_*i*_ denotes the measured variables (e.g., masses of solute and solvent), while *u*(*y*_*i*_) represents their corresponding standard uncertainties. The relative standard uncertainty was calculated as shown in eq 2. All uncertainty components were treated as standard uncertainties and combined assuming independent contributions. The resulting relative standard uncertainty in mole fraction solubility was estimated as *u*_r_(*x*_SA_) = 0.02.

2 $$u_{r}\left(x\right)=\frac{u\left(x\right)}{x}$$

## 3 Thermodynamic Framework

Thermodynamic relationships indicate that the equality of fugacities for a solid that partially dissolves in a liquid solvent can be expressed as shown in eq 3. In this equation, L represents the liquid phase, while S denotes the solid phase.

3 $${\mathrm{f̂}}_{i}^{S}={\mathrm{f̂}}_{i}^{L}$$

The fugacity of the solute in the liquid phase can be expressed using eq 4, where *f*_0_^L^ represents the fugacity of the pure solute in a subcooled liquid state, specifically below its melting point. In this context, *x*_*i*_ and γ_*i*_ denote the mole fraction and activity coefficient of the solute within the solution.

4 $${\mathrm{f̂}}_{i}^{S}=x_{i}\gamma_{i}f_{0}^{L}$$

Meanwhile, the fugacity of the solid remains equivalent to that of the pure solid as long as the solid phase is pure, allowing for the application of eq 5.

5 $${\mathrm{f̂}}_{i}^{S}=f_{0}^{S}$$

Combining eqs 3–5, the equilibrium conditions can be written as presented by eq 6.

6 $$x_{i}\gamma_{i}=\frac{f_{0}^{S}}{f_{0}^{L}}$$

Conversely, the ratio of fugacities (*f*_0_^S^/*f*_0_^L^) can be expressed using a thermodynamic relationship derived from the Gibbs–Helmholtz equation, assuming constant enthalpy of fusion and negligible heat capacity differences between phases. This leads to eq 7, where the melting point is denoted by the subscript F. The final equation, eq 8, can be derived by substituting eqs 7 into [Disp-formula eq6].

7 $$\ln\left(\frac{f_{0}^{S}}{f_{0}^{L}}\right)=\frac{\Delta H_{F}}{R}\left(\frac{1}{T_{F}}-\frac{1}{T}\right)$$

8 $$\ln\left(x_{i}\gamma_{i}\right)=\frac{\Delta H_{F}}{R}\left(\frac{1}{T_{F}}-\frac{1}{T}\right)$$

In addition to the enthalpy (Δ*H*_F_) and the temperature (*T*_F_) of fusion, calculating solubility (*x*) from eq 8 requires a thorough understanding of activity coefficients as a function of composition and temperature. These coefficients should be determined using suitable thermodynamic models, such as ASOG, PC-SAFT, or PSRK.

EoS, including PC-SAFT and PSRK, can also describe the activity coefficients, which are typically represented through an excess Gibbs energy method, as indicated in eq 9, where ϕ represents the fugacity coefficient.

9 $$\gamma_{i}^{L}=\frac{\phi_{i}^{L}}{\phi_{pure,i}^{L}}$$

## 4 Thermodynamic Models

### 4.1 Analytical Solutions of Groups

The ASOG model^29,30^ is a group contribution method for estimating activity coefficients in nonideal liquid mixtures. In accordance with eq 10, the activity coefficient of component *i* is expressed as the sum of a combinatorial term and a residual term.

10 $$\ln\gamma_{i}=\ln\gamma_{i}^{FH}+\ln\gamma_{i}^{R}$$

The combinatorial contribution, based on the Flory–Huggins approach, accounts for molecular size differences and depends on the mole fractions *x*_*i*_ and volume parameters *V*_*j*_ of the components, as described by eq 11.

11 $$\ln\gamma_{i}^{FH}=\ln\left(\frac{V_{i}}{\sum_{j}x_{j}V_{j}}\right)+1-\frac{V_{i}}{\sum_{j}x_{j}V_{j}}$$

The residual term, as presented by eq 12, accounts for group interactions and is expressed as a function of the number of occurrences ν_*k*,*i*_ of each group *k* in molecule *i*, as well as the group activity coefficients in the mixture, Γ_k_, and in the reference state of pure component *i*, Γ_k_^(*i*)^.

12 $$\ln\gamma_{i}^{R}=\sum_{k}\nu_{k,i}\left(\ln\Gamma_{k}-\ln\Gamma_{k}^{\left(i\right)}\right)$$

The group activity coefficient is calculated using a Wilson-type expression that depends on the group fractions *X*_*n*_ in the mixture and the interaction parameters Ψ_*k*,*n*_, as shown by eq 13.

13 $$\ln\Gamma_{k}=-\ln\left(\sum_{n}X_{n}\Psi_{n,k}\right)+1-\sum_{n}\frac{X_{n}\Psi_{k,n}}{\sum_{m}X_{m}\Psi_{m,n}}$$

The group fractions are obtained from the overall composition of the mixture, considering the mole fractions of each component and the number of occurrences of each functional group in its molecular structure. The interaction parameters between groups are temperature-dependent and are typically expressed as exponential functions of binary interaction energies, which may be asymmetric and described by empirical constants. These parameters are commonly correlated with temperature using linear or inverse-temperature relationships, allowing the model to capture thermal effects on intermolecular interactions.

The ASOG model is commonly applied to phase equilibrium calculations; however, its accuracy depends on the availability of reliable group interaction parameters, which in turn rely on high-quality experimental data.

### 4.2 PC-SAFT Equation of State

The Perturbed Chain Statistical Associating Fluid Theory (PC-SAFT) equation of state describes fluid-phase behavior by expressing the residual Helmholtz energy as the sum of three contributions associated with hard-chain repulsion, dispersion interactions, and molecular association,^31,32^ as in eq 14.

14 $$\frac{a^{res}}{RT}=\frac{a^{hc}}{RT}+\frac{a^{disp}}{RT}+\frac{a^{assoc}}{RT}$$

In this framework, non-associating components are characterized by three pure-component parameters, namely the number of segments *m*, the segment diameter σ, and the dispersion energy parameter ε, which represents the van der Waals attraction between segments. For associating components, two additional parameters are required, corresponding to the association energy $\varepsilon^{A_{i}B_{i}}$ and the association volume $\kappa^{A_{i}B_{i}}$ , which describe specific interactions such as hydrogen bonding.

For mixtures, cross-interaction parameters are obtained using combining rules,^33^ as presented by eqs 15 and 16. The segment diameter between components *i* and *j*, denoted as σ_*ij*_, is calculated as the arithmetic mean of the pure-component diameters, while the dispersion energy parameter ε_*ij*_ is obtained from the geometric mean corrected by a binary interaction parameter *k*_*ij*_.

15 $$\sigma_{ij}=\frac{1}{2}\left(\sigma_{i}+\sigma_{j}\right)$$

16 $$\varepsilon_{ij}=\left(1-k_{ij}\right)\sqrt{\varepsilon_{i}\varepsilon_{j}}$$

For associating systems, additional combining rules, eqs 17 and 18, are required to describe cross-association between sites belonging to different molecules. The cross-association energy between sites A_*i*_ and B_*j*_ is typically taken as the arithmetic mean of the self-association energies, while the cross-association volume is obtained from the geometric mean of the pure-component values combined with a size-dependent correction term.

17 $$\varepsilon^{A_{i}B_{j}}=\frac{1}{2}\left(\varepsilon^{A_{i}B_{i}}+\varepsilon^{A_{j}B_{j}}\right)$$

18 $$\kappa^{A_{i}B_{j}}=\sqrt{\kappa^{A_{i}B_{i}}\kappa^{A_{j}B_{j}}}\left[\frac{1}{2}\frac{\sqrt{\sigma_{ii}\sigma_{jj}}}{(\sigma_{ii}+\sigma_{jj}}\right]{}^{3}$$

The fugacity coefficient of component *i*, ${\mathrm{\phi ̂}}_{i}$ , is obtained from the residual Helmholtz energy and its composition derivatives, accounting for the dependence on temperature, volume, and mixture composition, leading to eq 19.

19 ln⁡ϕ̂i=AresnRT+(∂(AresnRT)∂xi)T,V,xk≠i−∑j=1N[xj(∂(AresnRT)∂xj)T,V,xk≠j]+Z−1−ln⁡Z

### 4.3 PSRK Equation of State

The PSRK (predictive Soave–Redlich–Kwong)^34^ equation of state combines the cubic SRK equation with a group contribution approach to describe nonideal mixtures, particularly when binary interaction parameters are unavailable or incomplete. This formulation incorporates excess Gibbs energy, typically obtained from a UNIFAC-type model, into the mixing rules, improving the prediction of phase equilibria in strongly nonideal systems.^35^

The distinguishing feature of the PSRK model is the use of a mixing rule that relates the attractive term of the equation of state to the excess Gibbs energy of the mixture, as shown by eq 20 in which *b* is the covolume parameter.

20 $$\frac{a}{bRT}=\sum_{i}x_{i}\frac{a_{i}}{b_{i}RT}+\frac{g^{E}}{RT}+\sum_{i}x_{i}\ln\left(\frac{b}{b_{i}}\right)$$

In this expression, *a* and *b* are the mixture parameters, *x*_*i*_ is the mole fraction of component *i*, and *g* ^E^ is the excess Gibbs energy at a reference pressure, typically calculated using a group contribution model such as UNIFAC. The parameters *a*_*i*_ and *b*_*i*_ correspond to the pure-component values derived from critical properties. The first equation, eq 21, represents the attractive interactions, and the second equation, eq 22, represents the repulsive interactions.

21 $$a_{i}=0.42748\frac{{\left({RT}_{C_{i}}\right)}^{2}}{P_{C_{i}}}\alpha_{i}\left(T\right)$$

22 $$b_{i}=0.08664\frac{RT_{C_{i}}}{P_{C_{i}}}$$

The temperature dependence of the attractive term is introduced through the function α_*i*_(*T*), which depends on the reduced temperature and empirical coefficients as described by eq 23, where the parameters *c*_1_, *c*_2_, and *c*_3_ are calculated in the generalized form of the acentric factor,^36^ as presented by eqs 24 and 25.

23 $$\alpha_{i}\left(T\right)={\left[1+c_{1}\left(1-\sqrt{\frac{T}{T_{C_{i}}}}\right)+c_{2}\left(1-\sqrt{\frac{T}{T_{C_{i}}}}\right)+c_{3}\left(1-\sqrt{\frac{T}{T_{C_{i}}}}\right)\right]}^{2}$$

24 $$c_{1}=0.48+1.574w_{i}-0.176w_{i}^{2}$$

25 $$c_{2}=c_{3}=0$$

The PSRK model has been widely applied to vapor–liquid equilibrium calculations, particularly for systems exhibiting strong nonideal behavior, and has also been successfully used to describe solid–liquid equilibrium.^37^ Its performance is strongly influenced by the accuracy of the excess Gibbs energy model and the quality of the underlying group interaction parameters.

### 4.4 Parameter Estimation

For the ASOG model, most group interaction parameters were obtained from the literature.^29^ However, the interaction parameters between the groups ArCH and H_2_O (*m*_k,l_, *m*_l,k_, *n*_k,l_, and *n*_l,k_) were treated as adjustable parameters and estimated from the experimental solubility data. The results are presented in Table 5, where all interaction parameters are explicitly classified according to their origin (literature-based or regressed in this work), thereby ensuring the full transparency and reproducibility of the modeling procedure.

The PSRK equation of state requires critical properties and acentric factors of pure components (taken from the literature unless otherwise stated). Since salicylic acid is a high-molecular-weight associating compound that undergoes thermal decomposition before reaching its critical region, experimental critical properties are not available in the literature.^38^ Therefore, the critical temperature, critical pressure, and acentric factor were estimated using group contribution methods.^39,40^

In this work, the PSRK model was implemented by using the UNIFAC-Dortmund approach within its mixing rule. Although the model is based primarily on transferable group contribution parameters, its reliability depends on the availability of interaction parameters between the functional groups present in the mixture. Since some UNIFAC-Dortmund interaction parameters (9–14, 15–17, and 16–17), corresponding to ACH–OH, CH_3_OH–ACOH, and H_2_O–ACOH subgroup interactions in the UNIFAC-Dortmund matrix defined in Table 9, were unavailable, they were treated as adjustable parameters and estimated from the experimental data of the present study prior to the PSRK calculations. This procedure allowed the PSRK model to be applied to the investigated systems within a group contribution framework.

For the PC-SAFT equation of state, the pure-component parameters (*m*, σ, ε/*k*, $k^{A_{i}B_{i}}$ , and $\varepsilon^{A_{i}B_{i}}/k$ ) were determined by fitting vapor pressure and saturated liquid density values obtained from the DIPPR database (DIPPR-based fitted correlations).^41^ Equations S1–S3 and the parameters employed in these calculations (Tables S1 and S2) are provided in the Supporting Information. Subsequently, the binary interaction parameter *k*_*ij*_, associated with the dispersion energy contribution, was adjusted to the experimental phase equilibrium (solubility) data. All binary interaction parameters were regressed within the same objective function framework described for the other models. To capture temperature effects, *k*_*ij*_ was expressed as a temperature-dependent function.

All adjustable parameters, regardless of the model, were estimated by minimizing a single objective function based on the modified maximum likelihood method. This unified approach ensures internal consistency and enables a direct comparison of model performance, considering 465 experimental mole fraction data points simultaneously. The objective function applied is given by eq 26, where *i* represents the components and *j* the experimental data points. The superscripts EXP and CALC denote experimental and calculated values, respectively.

26 $$OF=\sum_{i}\sum_{j}{\left(\ln\gamma_{i}^{\mathrm{EXP}}-\ln\gamma_{i}^{CALC}\right)}_{j}^{2}$$

The performance of the models was quantitatively assessed through the average relative deviation (ARD %) and the root-mean-square deviation (RMSD) between the experimental and calculated compositions. While PC-SAFT and ASOG were evaluated in a correlative framework, PSRK was evaluated using a reduced-parameter group contribution approach. This comparison highlights the different balances between transferability and parameter adjustment adopted by the models. These metrics are defined by eqs 27 and 28, where *x* represents the mole fraction, and *N* is the total number of experimental data points.

27 $$ARD\;\%=\frac{100}{N}\sum_{i=1}^{N}\frac{\left|x_{i}^{\mathrm{EXP}}-x_{i}^{CALC}\right|}{x_{i}^{\exp}}$$

28 $$RMSD=\sqrt{\frac{\sum_{i=1}^{N}{\left(\ln x_{i}^{\mathrm{EXP}}-\ln x_{i}^{CALC}\right)}^{2}}{N}}$$

In this work, different modeling strategies were intentionally adopted to highlight differences in the level of parametrization among the thermodynamic approaches. PC-SAFT and ASOG were used within a correlative framework, in which model parameters were regressed to experimental solubility data to evaluate their maximum descriptive capability. In contrast, the PSRK model was employed using predominantly transferable group contribution parameters, complemented by a limited number of estimated interaction parameters. This strategy enables a consistent comparison of the ability of the models to represent phase behavior across different levels of parametrization.

To ensure full transparency and reproducibility of the thermodynamic modeling framework, a comprehensive summary of the origin and estimation strategy of all parameters employed in this work is provided in the Supporting Information (Table S3). This classification explicitly distinguishes between literature-based, estimated, and regressed parameters across all models, allowing a clear identification of the level of parametrization adopted in each approach. This unified representation ensures that all calculations can be independently reproduced and that the methodological differences between correlative and reduced-parameter (group contribution) models are fully traceable.

## 5 Results and Discussion

### 5.1 Experimental Data

Table 4 compares the experimental solubility data for salicylic acid in pure solvents to the corresponding values reported in the literature. For all investigated systems, solubility increases monotonically with temperature, which is characteristic of an endothermic dissolution process in which thermal energy favors the disruption of intermolecular interactions within the crystalline phase and enhances solute–solvent interactions. The experimental trends are in satisfactory agreement with the published data, indicating the thermodynamic consistency of the results obtained.

In water, the absolute deviations between the experimental and literature values typically range from 1 × 10^–5^ to 1 × 10^–4^ in mole fraction, corresponding to relative deviations of 3% to 16%. These deviations can be partially attributed to the high sensitivity of salicylic acid solubility in highly polar media, which can be partially attributed to the strong hydrogen-bonding network of water, which acts as an antisolvent by reducing the availability of solvent molecules for effective solvation of salicylic acid, making the system highly sensitive to small variations in temperature and composition.

For alcohols, a higher degree of quantitative agreement with the literature data is observed when compared to the aqueous system, which can be attributed to the reduced structural complexity of the hydrogen-bonding network in alcohol-containing mixtures compared to pure water. In methanol, absolute deviations remained below 2 × 10^–3^ over the entire temperature range, with relative deviations generally lower than 1.1%, indicating good consistency of the experimental measurements. In the case of ethanol, although the temperature dependence is fully reproduced, greater dispersion among published data is observed at higher temperatures, with relative deviations reaching up to 5%. This can be associated with temperature-induced changes in the hydrogen-bonding structure of the solvent mixture. As a result, the experimental values obtained in this work lie between or very close to the extreme values reported in the literature.

Although solubility data for salicylic acid in pure 2-propanol are available in the literature, the reported values are limited and scattered, justifying the acquisition of new, consistent experimental data. Overall, the observed deviations fall within the typical range of experimental uncertainty for solubility measurements, confirming the reliability of the data generated in this study.

Figure 1 illustrates the solubility behavior of salicylic acid in binary liquid mixtures as a function of the mole fraction of water at different temperatures.

**Figure 1:**
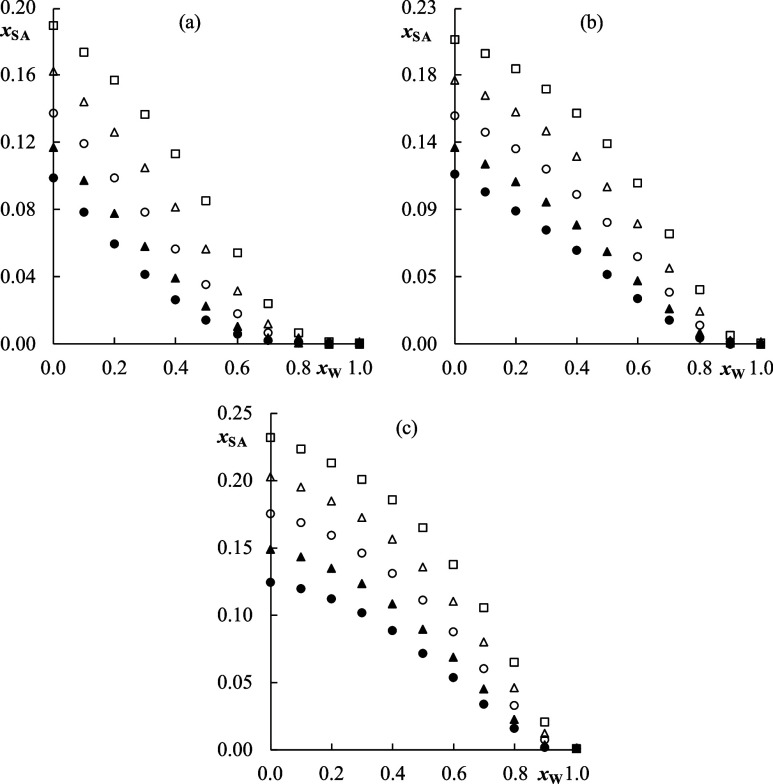
Solubility of salicylic acid (*x*_SA_) as a function of water mole fraction (*x*_W_) in the solutions of (a) water + methanol, (b) water + ethanol, and (c) water +2-propanol: **●** 283.2 K; ▲ 293.2 K; ○ 303.2 K; Δ 313.2 K; □ 323.2 K.

The experimental results indicate that the solubility of salicylic acid in aqueous–alcoholic mixtures is strongly influenced by both the temperature and the nature of the alcohol present in the solvent. In all of the studied systems, an increase in the solute mole fraction is observed with increasing temperature, which is consistent with the temperature dependence expected for solid–liquid equilibrium systems. This behavior reflects enhanced solvation of the solute at higher temperatures.

The addition of water plays a decisive role in the solubility of salicylic acid. As the mole fraction of water increases, a pronounced reduction in solubility is observed for all of the evaluated systems, particularly in water-rich compositions. The sharp decrease in solubility with increasing water fraction is consistent with the increase in solvent polarity and the strengthening of the hydrogen-bond network characteristic of water, which acts as an antisolvent for salicylic acid, thereby reducing the solvation efficiency of the relatively nonpolar aromatic ring.

In addition to the thermal effect, the type of alcohol used in the binary mixture significantly influences the salicylic acid solubility. At the same temperature, systems containing alcohols with lower dielectric constants exhibit higher solubility values. The partial substitution of water by alcohol reduces the dielectric constant of the solution and improves compatibility between the solvent medium and the hydrophobic character of salicylic acid, highlighting the important role of solvent composition in controlling the solid–liquid equilibrium.

The dielectric constant is used here solely as a qualitative descriptor of the solvent polarity. The thermodynamic behavior is primarily governed by molecular-level interactions rather than macroscopic polarity descriptors. The observed trends are primarily dictated by specific solute–solvent interactions, particularly associative and hydrophobic effects. The progressive decrease in the dielectric constant with an increasing alcohol fraction reduces the polarity of the medium and favors solute–solvent interactions compatible with the hydrophobic aromatic structure of salicylic acid. This effect becomes more pronounced for alcohols with longer carbon chains, such as 2-propanol, due to the increased hydrophobic character of the solvent, which enhances favorable interactions with the aromatic moiety of salicylic acid and reduces the relative strength of solute–solute interactions within the solid phase.

Furthermore, the influence of solvent composition becomes more pronounced at higher temperatures, suggesting a combined effect of thermal energy and solvent polarity on the solvation process. Under these conditions, solute–solvent interactions become increasingly favorable, particularly in less-polar media.

The results obtained are consistent with the trends reported in the literature^37,42^ for poorly soluble aromatic compounds in aqueous–alcoholic mixtures, supporting the interpretation based on solvent polarity and intermolecular interactions. The experimental solubility data related to this article are included in the Supporting Information as Table S4.

### 5.2 Modeling Results

For the ASOG model, the interaction parameters between the groups ArCH and H_2_O were estimated in this work using experimental solubility data, whereas the remaining parameters were taken from the literature.^29^ The results are presented in Table 5.

Table 6 presents the pure component parameters of the PC-SAFT EoS, obtained by fitting vapor pressure and liquid density values generated from the DIPPR database according to the methodology previously reported.^25,43^ For comparison, pharmaceutical compounds with very low volatility may also have their parameters estimated by fitting solubility data, as discussed elsewhere.^44^

Table 7 presents the temperature dependence of the binary interaction parameters for the systems involving salicylic acid, water, methanol, ethanol, and 2-propanol obtained using PC-SAFT. The parameters are valid within the experimental temperature range (283.15 K ≤ *T* ≤ 323.15 K).

Tables 9 and 10 present, respectively, the subgroup coefficients and the binary interaction parameters of the UNIFAC-Dortmund model applied to calculate the excess Gibbs energy. The binary interaction parameters *a*_mn_, *a*_nm_, *b*_mn_, and *b*_nm_ for interactions between subgroups 9–14, 15–16, and 16–17 were estimated in this study using experimental solubility data, yielding a total of six new parameters.

**Table 9:** Selected Standard UNIFAC-Dortmund Group Parameters Employed in the Present Work

functional group	description	*R*_k_	*Q*_k_
CH_3_	alkyl group	0.9011	0.8480
CH_2_	alkyl group	0.6744	0.5400
CH	alkyl group	0.4469	0.2280
ACH	aromatic carbon	0.5313	0.4000
OH	hydroxyl group	1.0000	1.2000
CH_3_OH	methanol group	1.4311	1.4320
H_2_O	water group	0.9200	1.4000
ACOH	aromatic hydroxyl group	0.8952	0.6800

**Table 10:** UNIFAC-Dortmund Group Interaction Parameters (Literature and Fitted Parameters)^a^

*m*	*n*	*a*_*mn*_ (K)	*b*_*mn*_	*c*_*mn*_ (K^–1^)	*a*_*nm*_ (K)	*b*_*nm*_	*c*_*nm*_ (K^–1^)
1	2	86.02	0.0000	0.0000	–35.36	0.0000	0.0000
1	3	61.13	0.0000	0.0000	–11.12	0.0000	0.0000
1	9	476.4	0.0000	0.0000	26.76	0.0000	0.0000
1	14	391.5	0.0000	0.0000	–30.48	0.0000	0.0000
1	15	255.7	0.0000	0.0000	65.33	0.0000	0.0000
1	16	206.6	0.0000	0.0000	–83.98	0.0000	0.0000
1	17	920.7	0.0000	0.0000	1139.0	0.0000	0.0000
2	3	38.81	0.0000	0.0000	3.46	0.0000	0.0000
2	9	182.6	0.0000	0.0000	42.9	0.0000	0.0000
2	14	240.9	0.0000	0.0000	1.16	0.0000	0.0000
2	15	163.9	0.0000	0.0000	–28.7	0.0000	0.0000
2	16	61.11	0.0000	0.0000	–25.4	0.0000	0.0000
2	17	749.3	0.0000	0.0000	2000.0	0.0000	0.0000
3	9	25.77	0.0000	0.0000	140.1	0.0000	0.0000
3	14	161.7	0.0000	0.0000	–44.9	0.0000	0.0000
3	15	122.8	0.0000	0.0000	–22.3	0.0000	0.0000
3	16	90.49	0.0000	0.0000	–223.9	0.0000	0.0000
3	17	648.2	0.0000	0.0000	247.5	0.0000	0.0000
9*	14*	–190.4	0.0000	0.0000	412.6	0.0000	0.0000
9	15	–174.2	0.0000	0.0000	394.6	0.0000	0.0000
9	16	–169.0	0.0000	0.0000	225.3	0.0000	0.0000
9	17	6201.0	0.0000	0.0000	–450.3	0.0000	0.0000
14	15	–107.2	0.0000	0.0000	127.4	0.0000	0.0000
14	16	–41.11	0.0000	0.0000	38.9	0.0000	0.0000
14	17	–200.7	0.0000	0.0000	–15.1	0.0000	0.0000
15	16	–189.2	0.0000	0.0000	865.9	0.0000	0.0000
15*	17*	–171.5	0.0000	0.0000	784.3	0.0000	0.0000
16*	17*	–207.8	0.0000	0.0000	815.9	0.0000	0.0000

a Interaction parameters marked with an asterisk were estimated in this work using experimental phase equilibrium (solubility) data. All other parameters were taken from the standard UNIFAC-Dortmund database.^35^

The UNIFAC-Dortmund group parameters were obtained from the official UNIFAC-Dortmund parameter database maintained by DDBST GmbH (https://www.ddbst.com/psrk.html). The selected values correspond to the parameters reported in the literature.^35^

Table 11 summarizes the average relative deviations (ARD %) and root-mean-square deviations (RMSD) for the systems investigated in this study. Table S5, provided in Supporting Information, presents the composition of the ternary liquid mixture calculated by the thermodynamic models. Furthermore, Figures S1–S15 in the Supporting Information illustrate the performance of the applied models for each binary mixture and temperature studied.

**Table 11:** Average Relative Deviations and Root-Mean-Square Deviations Found in This Study

model	ARD %	RMSD
Salicylic Acid + Water + Methanol		
ASOG	9.9878	0.1331
PC-SAFT	3.7917	0.0470
PSRK	6.3911	0.1017
Salicylic Acid + Water + Ethanol		
ASOG	17.6403	0.2871
PC-SAFT	4.6231	0.1064
PSRK	9.4112	0.1927
Salicylic Acid + Water +2-Propanol		
ASOG	7.5045	0.1351
PC-SAFT	1.5128	0.0295
PSRK	2.5805	0.0531

The performance of the thermodynamic models can be interpreted in terms of their theoretical foundations, their ability to represent molecular interactions, and the level of parametrization employed. Within the correlative framework adopted in this study, PC-SAFT provided the most accurate description of the experimental data. However, it should be noted that while PC-SAFT and ASOG rely on adjustable binary interaction parameters, PSRK was applied using predominantly transferable group contribution parameters and a substantially lower degree of parametrization than PC-SAFT and ASOG, which naturally affects the direct comparison of model performance.

Although PC-SAFT involves parameter adjustment, its superior performance is also associated with its molecular-based formulation, which explicitly accounts for both the dispersion and association interactions. Consequently, the model provides a more accurate representation of hydrogen bonding and size/shape effects in strongly nonideal mixtures.

In contrast, the ASOG model exhibited the highest deviations, particularly in aqueous-rich compositions, reflecting the limitations of group contribution methods in describing highly specific intermolecular interactions and local structural effects in aqueous systems. Although a limited number of parameters were adjusted in this work, the transferable nature of group-based approaches restricts their ability to accurately represent the complex balance between polar and nonpolar interactions present in these mixtures.

The largest deviations with ASOG were observed in the aqueous-rich region of the ethanol–water system, likely due to hydrophobic hydration effects, where the aromatic ring of salicylic acid promotes the formation of a more structured water network around the solute. Group contribution methods, which are based on the additivity of functional groups, typically underestimate these specific structural effects, resulting in the observed ARD values.

The PSRK model showed intermediate performance, despite relying predominantly on transferable group contribution parameters and requiring a considerably lower level of parameter adjustment than the correlative approaches. Its satisfactory results can be partially attributed to the incorporation of excess Gibbs energy through the UNIFAC-Dortmund model, which enhances the description of nonideal behavior. However, its accuracy is still influenced by the availability and quality of the underlying group contribution parameters and, when applicable, the estimation of required pure-component properties within the employed framework, which defines its domain of applicability for complex associating systems. These aspects are inherent to group contribution-based thermodynamic frameworks and define their applicability range rather than limiting their general validity.

Overall, the results indicate that model performance is governed not only by the number of adjustable parameters but also, more importantly, by the ability of the model to represent the underlying physical interactions. These findings highlight the importance of balancing model complexity, parametrization strategy, and model applicability while also emphasizing the potential of reduced-parameter thermodynamic approaches such as PSRK for engineering applications where experimental data are scarce.

Among the three investigated solvent systems, the ethanol-containing mixtures consistently showed the highest deviations across all of the thermodynamic models. This behavior can be attributed to the intermediate polarity of ethanol, which leads to a delicate balance between hydrogen-bonding interactions involving the hydroxyl group and hydrophobic interactions associated with its ethyl chain. As a result, ethanol promotes complex local solvent structuring in aqueous–alcoholic mixtures, where competing solute–solvent and solvent–solvent interactions become more pronounced. These effects are particularly difficult to capture quantitatively using the thermodynamic frameworks employed, leading to systematically higher deviations compared with methanol and 2-propanol-containing mixtures.

### 5.3 Performance of Thermodynamic Models

Although PC-SAFT and ASOG were applied in a correlative framework through adjustment of binary interaction parameters, the PSRK model was employed within a group contribution framework based primarily on UNIFAC parameters with only a limited number of system-specific interaction parameters.

The performance of PSRK was evaluated by comparing the calculated and experimental solubility trends across the three solvent systems. Despite the reduced level of parametrization, the model successfully reproduces the main qualitative features of the systems, including the monotonic decrease in solubility with increasing water mole fraction and the correct relative order of solubility among the studied alcohols (methanol > ethanol >2-propanol).

This agreement indicates that PSRK captures the dominant thermodynamic driving forces governing the phase equilibrium. However, quantitative deviations are observed, particularly in aqueous-rich compositions, where strong associative interactions and local structuring effects become more pronounced and are less accurately represented by group contribution-based excess Gibbs energy models.

Despite these limitations, the model provides a physically consistent description of the systems and demonstrates its usefulness in supporting solvent trend analysis when experimental data are limited.

The performance of the PSRK framework is further supported by the quantitative deviation metrics (ARD % and RMSD), confirming that PSRK is capable of reproducing the main thermodynamic trends of the investigated systems while maintaining a limited level of system-specific parametrization.

## 6 Conclusion

This study provides new mole fraction solubility data for salicylic acid in binary mixtures of water with methanol, ethanol, and 2-propanol over the complete composition range and temperatures from 283.2 to 323.2 K at atmospheric pressure. The gravimetric method proved to be reliable, and the solubility data obtained for pure solvents showed satisfactory agreement with literature values, supporting the consistency and accuracy of the measurements.

The solubility increases monotonically with temperature in all investigated systems, confirming the endothermic nature of the dissolution process. Solvent composition strongly affects solubility, with an increasing water mole fraction leading to a pronounced decrease in solubility across all mixtures, confirming the antisolvent effect of water. This behavior is associated with the high polarity of water and its strong intermolecular interactions.

In contrast, the presence of alcohols promoted solubilization by providing a more favorable solvation environment for salicylic acid. The stronger cosolvent effect observed for less-polar alcohols is consistent with the improved compatibility between the solvent hydrophobicity and the aromatic structure of the solute. Furthermore, the magnitude of this phenomenon increased with the temperature, likely due to reduced solvent organization and enhanced molecular mobility.

From a thermodynamic modeling perspective, the evaluated approaches exhibited distinct capabilities in representing experimental data. PC-SAFT showed the lowest deviations among the evaluated models within the correlative framework, yielding the lowest deviations, which can be attributed to its molecular-based formulation capable of explicitly accounting for dispersion and association interactions. The PSRK model, despite requiring substantially fewer adjusted parameters than the correlative approaches, showed satisfactory accuracy, highlighting its potential for screening and preliminary engineering applications, where experimental data are limited. In contrast, the ASOG model exhibited higher deviations, particularly in aqueous-rich compositions, reflecting the limitations of group contribution methods in describing highly specific intermolecular interactions.

By covering the complete composition range for three aqueous–alcoholic systems, this work expands the available solubility database for salicylic acid and provides a consistent experimental benchmark for thermodynamic modeling approaches. The results are relevant for solvent selection, antisolvent crystallization, and separation processes involving salicylic acid while also contributing to the understanding of solute–solvent interactions in mixed solvent systems.

## Supplementary Material



## Notes

Equations and parameters used to calculate vapor pressure and density; origin and determination methodology of the parameters used in the thermodynamic models; experimental solubility data for the studied mixtures at different temperatures; composition of the ternary liquid mixture calculated by the thermodynamic modeling; and visual representations showing the performance of the models (PDF)
