# Supplementary material for: Solubility Behavior of Salicylic Acid in Aqueous Alcohol Mixtures: Interplay of Temperature, Solvent Composition, and Thermodynamic Modeling
Source: ACS Omega. 2026 Jul 24;11(31):46583–94. doi: 10.1021/acsomega.6c04286 (PMC13470819; doi:10.1021/acsomega.6c04286)
Supplement: Supplementary file 1 [file ao6c04286_si_001.pdf]

Supporting Information

Solubility Behavior of Salicylic Acid in Aqueous Alcohol Mixtures: Interplay of Temperature, Solvent Composition, and Thermodynamic Modeling

Alessandro C. Galvão*[a], Guilherme A. Mariane[a], Raquel Bordignon[a], Igor G. Kaiser[a], Pedro F. Arce[b], Weber S. Robazza[a]

[a]*Laboratory ApTher - Applied Thermophysics, Department of Food and Chemical Engineering, Santa Catarina State University - UDESC, 89870-000, Pinhalzinho-SC, Brazil*

[b]*Laboratory of Applied Thermodynamics and Separation Processes, Department of Chemical Engineering, Engineering School of Lorena, University of São Paulo - USP, 12602-810, Lorena-SP, Brazil*

*Corresponding author: alessandro.galvao@udesc.br

The vapor pressure P^{sat}(Pa) of the components used in this work as a function of temperature T(K) was calculated in accordance with Eq. (S1). The parameters of the equation are described in Table S1.

$$P^{\text{sat}} = \exp\left(\mathrm{A} + \frac{\mathrm{B}}{T} + \mathrm{C}\ln T + \mathrm{D}T^{\mathrm{E}}\right) \tag{S1}$$

Table S1. Parameters [1] of the Eq. (S1) applied to calculate the vapor pressure

	A	B	C	D	E
water	73.649	-7258.2	-7.3037	4.1653×10^{-6}	2
methanol	82.718	-6904.5	-8.8622	7.4664×10^{-6}	2
ethanol	73.304	-7122.3	-7.1424	2.8853×10^{-6}	2
2-propanol	76.430	-7607.0	-7.4086	4.3986×10^{-18}	6
salicylic acid	388.890	-25362.0	-56.1790	4.3257×10^{-2}	1

The density ρ(kmol·m^{-3}) of the components used in this work as a function of temperature T(K) was calculated in accordance with Eq. (S2) for methanol, ethanol, 2-propanol, and salicylic acid and Eq. (S3) for water.

$$\rho = \frac{\mathrm{A}}{\mathrm{B}^{\left(1+\left(1-\frac{T}{\mathrm{C}}\right)^{\mathrm{D}}\right)}} \tag{S2}$$

$$\rho = A + B(1-T_r)^{0.35} + C(1-T_r)^{2/3} + D(1-T_r) + E(1-T_r)^{4/3} \tag{S3}$$

Table S2. Parameters [1] of the Eq. (S2) and Eq. (S3) applied to calculate the density

	A	B	C	D	E
water	17.863	58.606	-95.396	213.89	-141.26
methanol	2.32670	0.27073	512.5	0.24713	-
ethanol	1.62880	0.27469	514.0	0.23178	-
2-propanol	1.18980	0.26648	508.3	0.23986	-
salicylic acid	0.84309	0.27520	739.0	0.28571	-

Table S3. Origin and determination methodology of the parameters used in the thermodynamic models

Model	Parameter type	Parameters	Origin	Method
PSRK (UNIFAC-Dortmund)	Group volume & surface	R_{k}, Q_{k}	Literature [2]	UNIFAC-Dortmund database
PSRK (UNIFAC-Dortmund)	Group interaction parameters	a_{mn}, a_{nm}, b_{mn}, b_{nm}, c_{mn}, c_{nm}	Literature [2]	UNIFAC-Dortmund database
PSRK (UNIFAC-Dortmund)	Fitted group interaction parameters	9-14, 15-17, 16-17	This work	Regression to experimental phase equilibrium (solubility) data
PSRK	Critical properties	T_{C}, P_{C}, ω	Literature [1] + Estimated [3,4]	Group contribution methods
ASOG	Group interaction parameters	$m_{\mathrm{k,l}}$, $m_{\mathrm{l,k}}$, $n_{\mathrm{k,l}}$, $n_{\mathrm{l,k}}$	Literature [5]	ASOG database
ASOG	Fitted group interaction parameters	ArCH-H_2O	This work	Regression to experimental phase equilibrium (solubility) data
PC-SAFT	Fitted pure-component parameters	m, σ, ε/k, $k^{\mathrm{A_iB_i}}$, $\varepsilon^{\mathrm{A_iB_i}}/k$	This work	Regression using vapor pressure and liquid density (DIPPR data)
PC-SAFT	Fitted binary interaction parameters	k_{ij}	This work	Regression to experimental phase equilibrium (solubility) data

Table S4. Solubility of salicylic acid expressed as a molar fraction (x_{SA}) at different temperatures as a function of water molar fraction (x_1) in the binary solvent mixture water (1) + methanol (2), water (1) + ethanol (2), and water (1) + 2-propanol (2) at 95.2 kPa ± 0.3 kPa

x_1	x_{SA}				
	283.2 K	293.2 K	303.2 K	313.2 K	323.2 K
		water (1) + methanol (2)			
1.0000	0.00015	0.00021	0.00032	0.00047	0.00070
0.9000	0.00016	0.00026	0.00044	0.00088	0.00172
0.8000	0.00049	0.00094	0.00175	0.00344	0.00679
0.7000	0.00197	0.00360	0.00645	0.01232	0.02403
0.5999	0.00574	0.01033	0.01774	0.03134	0.05436
0.5000	0.01403	0.02248	0.03562	0.05629	0.08570
0.4000	0.02631	0.03905	0.05691	0.08164	0.11316
0.3001	0.04176	0.05801	0.07880	0.10522	0.13692
0.2001	0.05960	0.07769	0.09932	0.12602	0.15694
0.1003	0.07881	0.09749	0.11910	0.14476	0.17413
0.0000	0.09918	0.11712	0.13795	0.16232	0.18976
		water (1) + ethanol (2)			
1.0000	0.00015	0.00021	0.00032	0.00047	0.00070
0.9000	0.00035	0.00061	0.00130	0.00290	0.00609
0.8001	0.00442	0.00766	0.01316	0.02242	0.03749
0.7000	0.01619	0.02404	0.03552	0.05196	0.07564
0.6000	0.03134	0.04348	0.06007	0.08218	0.11048
0.5000	0.04797	0.06326	0.08304	0.10782	0.13726
0.4004	0.06407	0.08147	0.10286	0.12869	0.15810
0.3003	0.07858	0.09764	0.11997	0.14571	0.17490
0.2005	0.09172	0.11126	0.13382	0.15953	0.18836
0.1003	0.10402	0.12343	0.14550	0.17037	0.19881
0.0000	0.11628	0.13473	0.15621	0.18119	0.20850
		water (1) + 2-propanol (2)			
1.0000	0.00015	0.00021	0.00032	0.00047	0.00070
0.9000	0.00172	0.00335	0.00691	0.01214	0.02094
0.8000	0.01572	0.02253	0.03254	0.04576	0.06447
0.7001	0.03398	0.04496	0.06056	0.07980	0.10534
0.6002	0.05330	0.06844	0.08748	0.11050	0.13791
0.5000	0.07170	0.08970	0.11149	0.13632	0.16500
0.4002	0.08826	0.10807	0.13133	0.15703	0.18556
0.3004	0.10179	0.12321	0.14671	0.17240	0.20084
0.2005	0.11238	0.13502	0.15920	0.18516	0.21362
0.1001	0.11988	0.14341	0.16875	0.19553	0.22410
0.0000	0.12407	0.14892	0.17538	0.20338	0.23260

Standard uncertainties u are $u(T)$ = 0.1 K; $u(x_1)$ = 0.0001; $u_r(x_{SA})$ = 0.02

Table S5. Composition of the ternary liquid mixture calculated by the thermodynamic modeling

ASOG		PC-SAFT		PSRK	
x_{SA}	x_2/x_3	x_{SA}	x_2/x_3	x_{SA}	x_2/x_3
salicylic acid (SA) + water (2) + methanol (3)					
283.2 K					
0.00115	1489.8	0.00032	19992.6	0.00019	19986.2
0.00012	8.8365	0.00018	9.0414	0.00021	8.9591
0.00064	3.9700	0.00053	4.0159	0.00061	3.9942
0.00251	2.3209	0.00185	2.3370	0.00213	2.2227
0.00650	1.4947	0.00542	1.4994	0.00616	1.4990
0.01513	0.99755	0.01328	1.0000	0.01442	0.99123
0.02741	0.66420	0.02565	0.66772	0.02691	0.66648
0.04317	0.42661	0.04113	0.42942	0.04238	0.42845
0.06093	0.24891	0.05881	0.25082	0.06012	0.24981
0.07982	0.10995	0.07812	0.11200	0.07923	0.11120
0.10112	0.00094	0.09844	0.00028	0.09968	0.00024
293.2 K					
0.00126	1636.3	0.00049	2561.8	0.00055	99944.0
0.00042	8.8656	0.00028	8.9415	0.00032	8.9709
0.00109	3.9746	0.00101	3.9830	0.00109	3.9935
0.00410	2.3233	0.00392	2.3297	0.00397	2.3322
0.01151	1.4942	0.01127	1.4996	0.01063	1.4984
0.02393	0.99634	0.02323	0.99904	0.02298	0.99992
0.04087	0.66571	0.03984	0.66596	0.03942	0.66608
0.07103	0.43428	0.05892	0.42826	0.05841	0.38624
0.07789	0.24800	0.07838	0.24932	0.07809	0.24964
0.09905	0.10983	0.09822	0.11111	0.09783	0.11098
0.11902	0.00105	0.11791	0.00106	0.11752	0.00053
303.2 K					
0.00054	24978.0	0.00064	4757.8	0.00064	99935.0
0.00061	8.9620	0.00047	8.9347	0.00055	8.9755
0.00203	3.9864	0.00183	3.9859	0.00193	3.9938
0.00716	2.3246	0.00721	2.3318	0.00684	2.3329
0.01857	1.4957	0.01786	1.5041	0.01812	1.4980
0.03662	0.99975	0.03668	1.0003	0.03609	0.99961
0.05814	0.66636	0.05753	0.66526	0.05732	0.66657
0.07967	0.42671	0.07932	0.42847	0.07925	0.42854
0.10081	0.24881	0.09996	0.24865	0.09982	0.25023
0.12067	0.10998	0.11992	0.11089	0.11956	0.11129
0.13910	0.00099	0.13865	0.00108	0.13822	0.00037
313.2 K					
0.00116	33293.7	0.00025	1085.7	0.00092	49953.0
0.00102	8.9164	0.00095	8.9368	0.00135	9.0035
0.00401	3.9882	0.00367	3.9894	0.00371	3.9957
0.01362	2.3310	0.01292	2.3319	0.01262	2.3316
0.03285	1.4991	0.03195	1.4977	0.03167	1.4985

0.05811	1.0007	0.05681	0.99845	0.05663	0.99930
0.08296	0.66595	0.08218	0.66601	0.08195	0.66645
0.10683	0.42827	0.10594	0.42848	0.10552	0.42843
0.12721	0.24929	0.12663	0.24942	0.12638	0.24962
0.14585	0.11079	0.14527	0.11083	0.14522	0.11119
0.16352	0.00110	0.16292	0.00106	0.16271	0.00026
323.2 K					
0.00146	3442.2	0.00027	99972.0	0.00106	49946.0
0.00192	8.9539	0.00159	9.0302	0.00192	8.9778
0.00735	3.9935	0.00622	4.0024	0.00717	4.0004
0.02511	2.3299	0.02337	2.3339	0.02443	2.3324
0.05597	1.4982	0.05381	1.5001	0.05471	1.4990
0.08693	0.99845	0.08526	1.0021	0.08615	1.0004
0.11497	0.66450	0.11273	0.66761	0.11353	0.66627
0.13802	0.42731	0.13638	0.42995	0.13726	0.42788
0.15804	0.24772	0.15642	0.25099	0.15722	0.25004
0.17532	0.10910	0.17376	0.11224	0.17449	0.11109
0.19118	0.00116	0.18934	0.00095	0.19012	0.00040
salicylic acid (SA) + water (2) + ethanol (3)					
283.2 K					
0.00126	1663.6	0.00032	33321.7	0.00085	24977.7
0.00154	8.9597	0.00057	9.0033	0.00092	8.9948
0.00531	3.9859	0.00461	3.9989	0.00506	4.0032
0.01798	2.3315	0.01648	2.3340	0.01692	2.3333
0.03283	1.4992	0.03153	1.4989	0.03191	1.4985
0.04982	1.0002	0.04838	0.99975	0.04878	0.99903
0.06568	0.66724	0.06442	0.66758	0.06484	0.66686
0.07952	0.42688	0.07886	0.42874	0.07927	0.42848
0.09309	0.24962	0.09191	0.25786	0.09233	0.24999
0.10531	0.11038	0.10435	0.11119	0.10468	0.11067
0.11730	0.00033	0.11662	0.00032	0.11693	0.00056
293.2 K					
0.00120	1848.6	0.00054	99945.0	0.00085	33304.0
0.00160	8.9760	0.00092	9.0088	0.00123	9.0147
0.00890	3.9894	0.00809	4.0026	0.00837	4.0019
0.02510	2.3324	0.02432	2.3326	0.02466	2.3322
0.04460	1.4959	0.04386	1.4999	0.04424	1.4983
0.06450	0.99804	0.06371	0.99998	0.06408	0.99983
0.08290	0.66633	0.08182	0.66702	0.08212	0.66672
0.09840	0.42812	0.09808	0.42867	0.09815	0.42813
0.11260	0.25011	0.11173	0.25033	0.11193	0.24994
0.12450	0.11059	0.12392	0.11113	0.12407	0.11071
0.13580	0.00056	0.13508	0.00040	0.13532	0.00057
303.2 K					
0.00136	2218.2	0.00065	49966.5	0.00094	99905.0
0.00222	8.9252	0.00169	9.0031	0.00192	8.9898

0.01425	3.9901	0.01363	4.0044	0.01391	4.0045
0.03681	2.3339	0.03591	2.3340	0.03617	2.3338
0.06157	1.4946	0.06047	1.4995	0.06064	1.4977
0.08424	0.99699	0.08343	0.99980	0.08363	0.99941
0.10408	0.66661	0.10338	0.66770	0.10357	0.66669
0.12132	0.42745	0.12032	0.42863	0.12074	0.42825
0.13485	0.24865	0.13414	0.25038	0.13442	0.24992
0.14671	0.11330	0.14587	0.11112	0.14608	0.11080
0.15728	0.00058	0.15651	0.00034	0.15682	0.00063
		313.2 K			
0.00154	5546.0	0.00085	99914.0	0.00108	99891.0
0.00402	8.9828	0.00322	9.0078	0.00343	8.9917
0.02397	4.0068	0.02278	4.0011	0.02296	3.9982
0.05341	2.3290	0.05243	2.3355	0.05261	2.3334
0.08364	1.4957	0.08262	1.5000	0.08285	1.4990
0.10882	0.99749	0.10816	0.99964	0.10835	0.99890
0.12995	0.66431	0.12914	0.66720	0.12962	0.66685
0.14693	0.42682	0.14603	0.42878	0.14637	0.42848
0.16102	0.24897	0.15988	0.25049	0.16028	0.25025
0.17217	0.11003	0.17072	0.11129	0.17105	0.11087
0.18303	0.00060	0.18151	0.00048	0.18192	0.00083
		323.2 K			
0.00165	4990.7	0.00113	49942.5	0.00132	7132.4
0.00803	9.0554	0.00662	9.0139	0.00687	9.0104
0.03901	4.0044	0.03798	3.9980	0.03813	3.9944
0.07709	2.3337	0.07607	2.3306	0.07628	2.3338
0.11203	1.5004	0.11092	1.4988	0.11113	1.4986
0.13822	0.99671	0.13771	0.99974	0.13809	0.99998
0.15997	0.66692	0.15869	0.66669	0.15883	0.66684
0.17618	0.42611	0.17543	0.42835	0.17569	0.42824
0.18992	0.24914	0.18894	0.24986	0.18912	0.24995
0.20058	0.10955	0.19921	0.11128	0.19964	0.11047
0.21041	0.00072	0.20916	0.00047	0.20928	0.00075
		salicylic acid (SA) + water (2) + 2-propanol (3)			
		283.2 K			
0.00137	2698.0	0.00045	9994.5	0.00074	99925.0
0.00303	8.9867	0.00201	8.9869	0.00232	9.0169
0.01702	3.9951	0.01604	3.9988	0.01637	4.0057
0.03525	2.3313	0.03432	2.3343	0.03466	2.3354
0.05451	1.4984	0.05368	1.5005	0.05403	1.5016
0.07309	1.0007	0.07204	0.99888	0.07248	0.99996
0.08943	0.66378	0.08862	0.66639	0.08902	0.66642
0.10302	0.42663	0.10218	0.42849	0.10257	0.42887
0.11386	0.24912	0.11272	0.25015	0.11319	0.25056
0.12122	0.11015	0.12048	0.11065	0.12061	0.11055

0.12568	0.00032	0.12459	0.00043	0.12473	0.00056
		293.2 K			
0.00164	8318.7	0.00043	6662.8	0.00073	49962.5
0.00551	9.0474	0.00362	8.9818	0.00381	8.9939
0.02418	4.0019	0.02289	3.9954	0.02309	4.0011
0.04667	2.3316	0.04553	2.3350	0.04554	2.3335
0.07051	1.5025	0.06882	1.4987	0.06893	1.4996
0.09114	0.99837	0.09028	1.0000	0.09032	0.99952
0.10952	0.66560	0.10854	0.66659	0.10866	0.66659
0.12488	0.42774	0.12365	0.42898	0.12389	0.42880
0.13633	0.24889	0.13559	0.25045	0.13553	0.25005
0.14492	0.10933	0.14382	0.11062	0.14392	0.11047
0.15038	0.00090	0.14936	0.00046	0.14946	0.00058
		303.2 K			
0.00164	9982.6	0.00083	49957.5	0.00109	99890.0
0.00825	8.9553	0.00741	9.0120	0.00763	9.0017
0.03392	3.9986	0.03297	3.9999	0.03326	4.0005
0.06217	2.3197	0.06103	2.3344	0.06131	2.3345
0.08919	1.4978	0.08795	1.5000	0.08814	1.5002
0.11302	0.99865	0.11192	0.99959	0.11228	0.99892
0.13294	0.66451	0.13198	0.66655	0.13204	0.66643
0.14802	0.42758	0.14715	0.42913	0.14748	0.42875
0.16116	0.24878	0.15961	0.25012	0.15993	0.25009
0.17032	0.10975	0.16917	0.11071	0.16942	0.11053
0.17697	0.00047	0.17593	0.00035	0.17628	0.00118
		313.2 K			
0.00205	99794.0	0.00093	99906.0	0.00123	4538.9
0.01409	9.0552	0.01256	8.9933	0.01287	8.9942
0.04721	3.9976	0.04622	4.0033	0.04651	3.9984
0.08126	2.3341	0.08038	2.3350	0.08052	2.3322
0.11223	1.4989	0.11091	1.5000	0.11208	1.5031
0.13817	0.99696	0.13686	0.99931	0.13704	0.99852
0.15828	0.66410	0.15744	0.66688	0.15783	0.66618
0.17393	0.42689	0.17282	0.42861	0.17317	0.42833
0.18718	0.24920	0.18566	0.25035	0.18593	0.24982
0.19703	0.10932	0.19594	0.11070	0.19626	0.11009
0.20461	0.00059	0.20392	0.00045	0.20401	0.00087
		323.2 K			
0.00213	19956.4	0.00115	49941.5	0.00142	49928.0
0.03137	9.8384	0.02132	8.9937	0.02166	8.9912
0.06591	3.9978	0.06507	4.0031	0.06521	3.9967
0.10658	2.3302	0.10594	2.3331	0.10607	2.3318
0.13924	1.4975	0.13833	1.5009	0.13863	1.4992
0.16662	0.99794	0.16548	0.99947	0.16578	0.99871
0.18708	0.66313	0.18603	0.66582	0.18632	0.66601

0.20233	0.42668	0.20131	0.42860	0.20151	0.42809
0.21495	0.24845	0.21416	0.25040	0.21437	0.24993
0.22552	0.10851	0.22457	0.11053	0.22484	0.11037
0.23418	0.00085	0.23305	0.00048	0.23336	0.00090

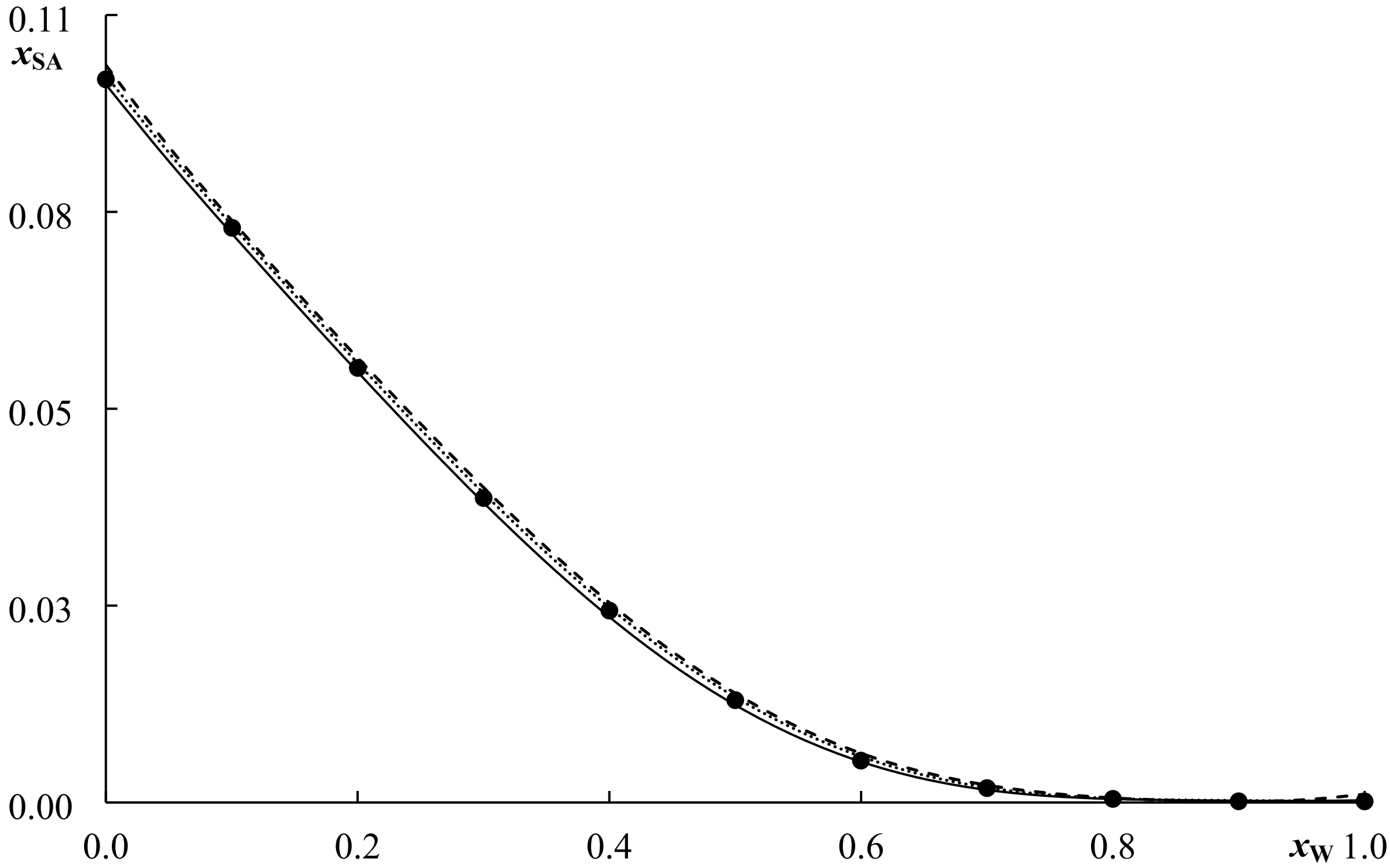

Figure S1. Solubility of salicylic acid (x_{SA}) as a function of water mole fraction (x_W) in the solutions of water + methanol at 283.2 K: ● experimental data; PC-SAFT is the continuous line; ASOG is the dashed line; PSRK is the doted line

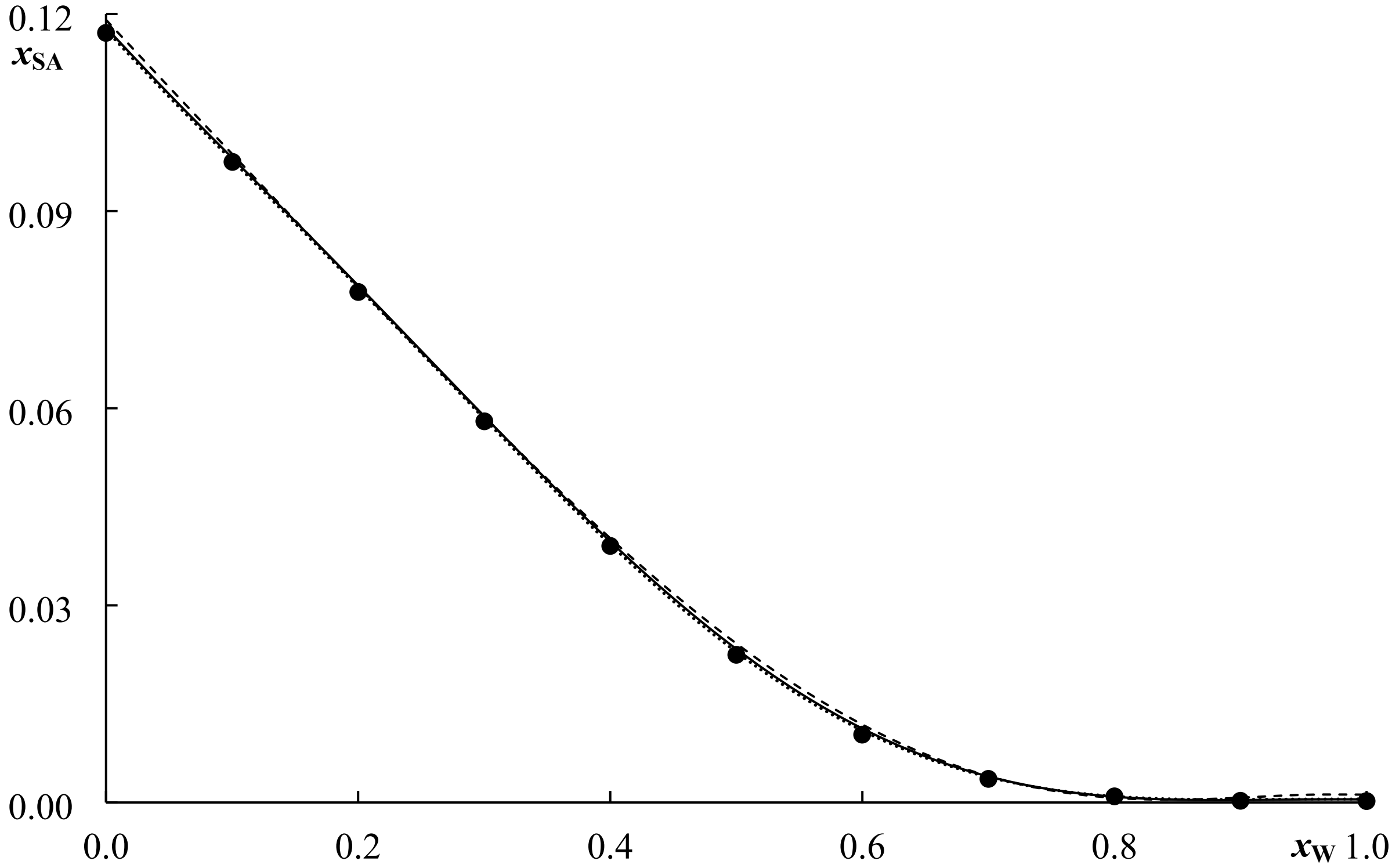

Figure S2. Solubility of salicylic acid (x_{SA}) as a function of water mole fraction (x_W) in the solutions of water + methanol at 293.2 K: ● experimental data; PC-SAFT is the continuous line; ASOG is the dashed line; PSRK is the doted line

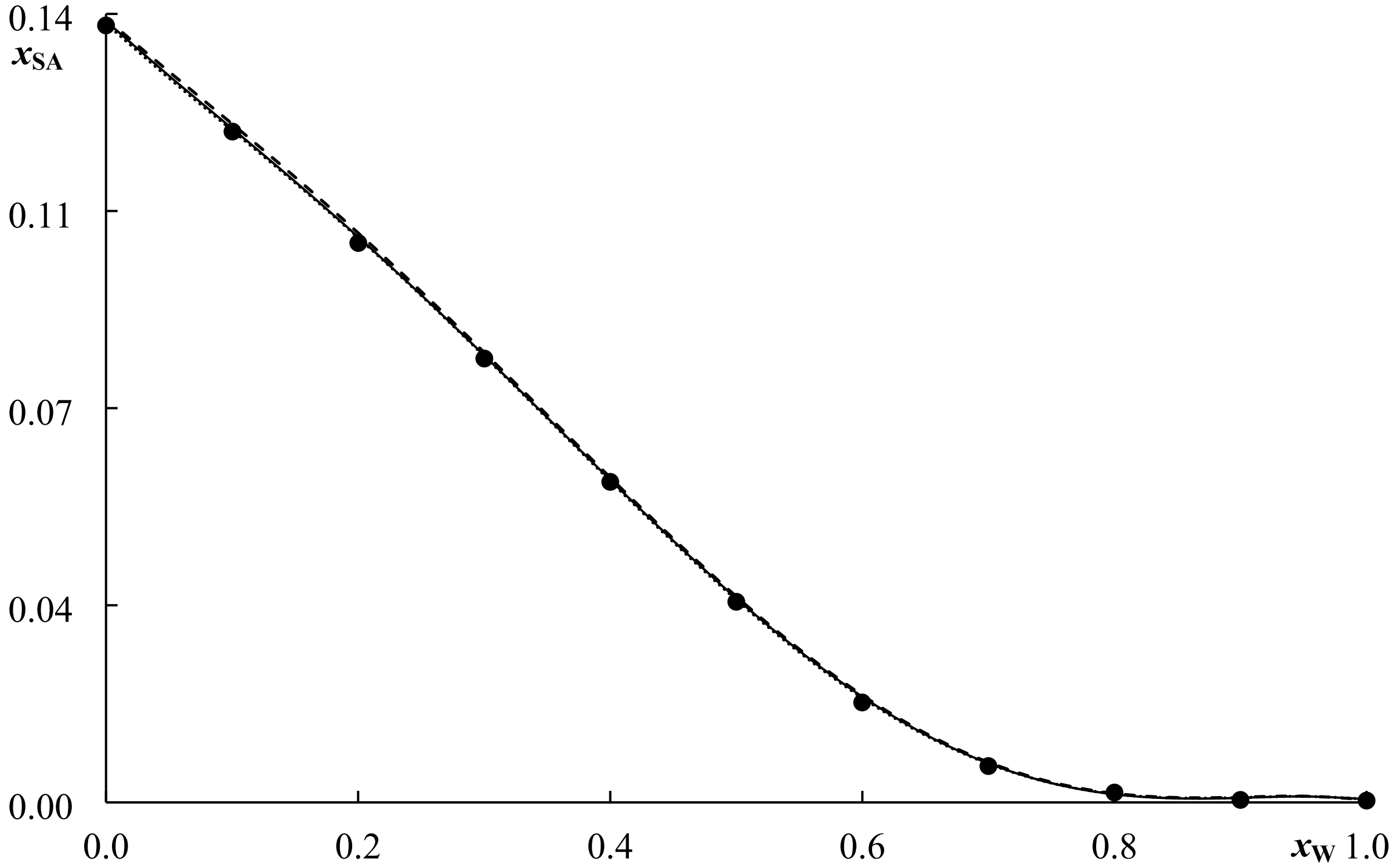

Figure S3. Solubility of salicylic acid (x_{SA}) as a function of water mole fraction (x_W) in the solutions of water + methanol at 303.2 K: ● experimental data; PC-SAFT is the continuous line; ASOG is the dashed line; PSRK is the doted line

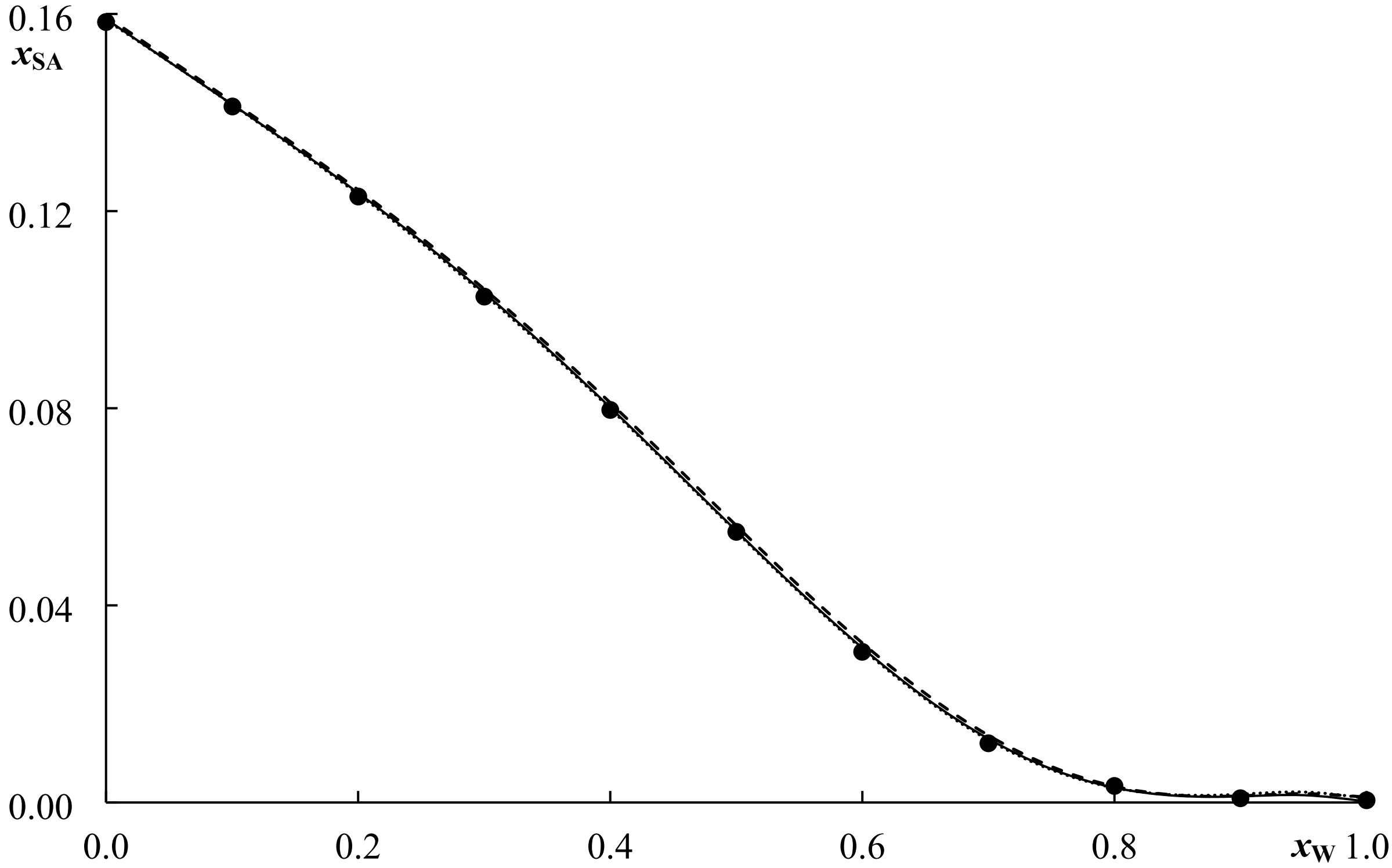

Figure S4. Solubility of salicylic acid (x_{SA}) as a function of water mole fraction (x_W) in the solutions of water + methanol at 313.2 K: ● experimental data; PC-SAFT is the continuous line; ASOG is the dashed line; PSRK is the doted line

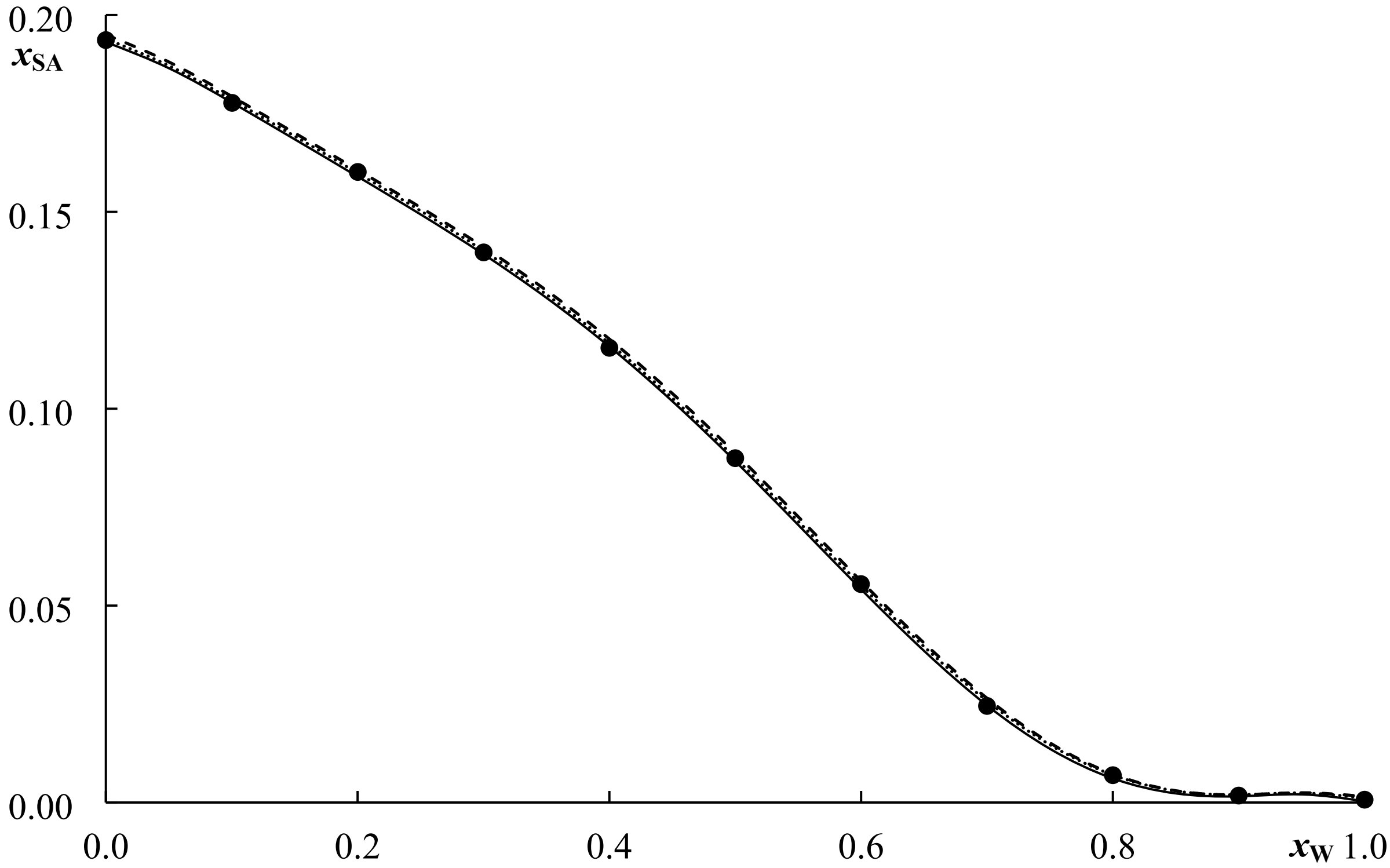

Figure S5. Solubility of salicylic acid (x_{SA}) as a function of water mole fraction (x_W) in the solutions of water + methanol at 323.2 K: ● experimental data; PC-SAFT is the continuous line; ASOG is the dashed line; PSRK is the doted line

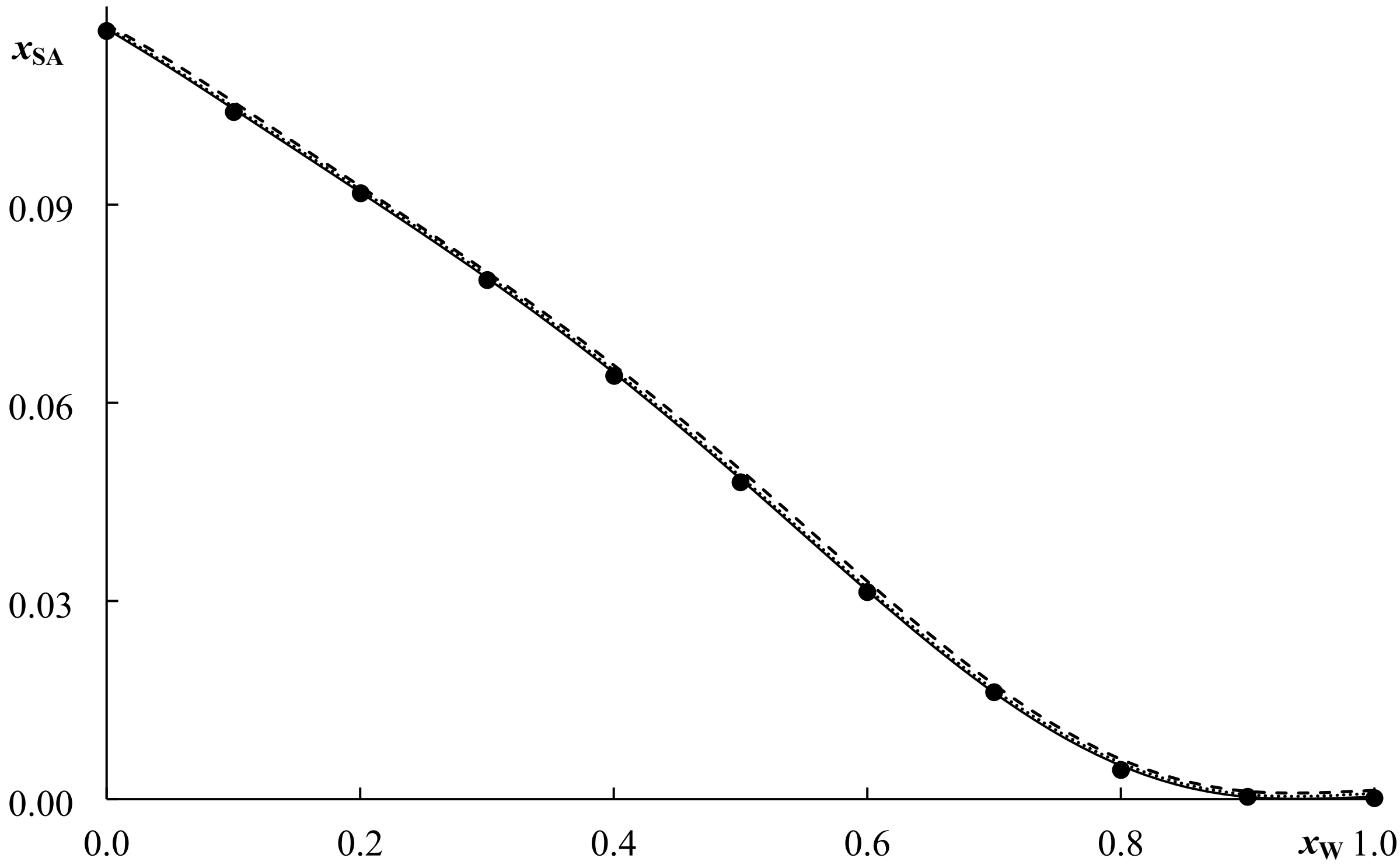

Figure S6. Solubility of salicylic acid (x_{SA}) as a function of water mole fraction (x_W) in the solutions of water + ethanol at 283.2 K: ● experimental data; PC-SAFT is the continuous line; ASOG is the dashed line; PSRK is the doted line

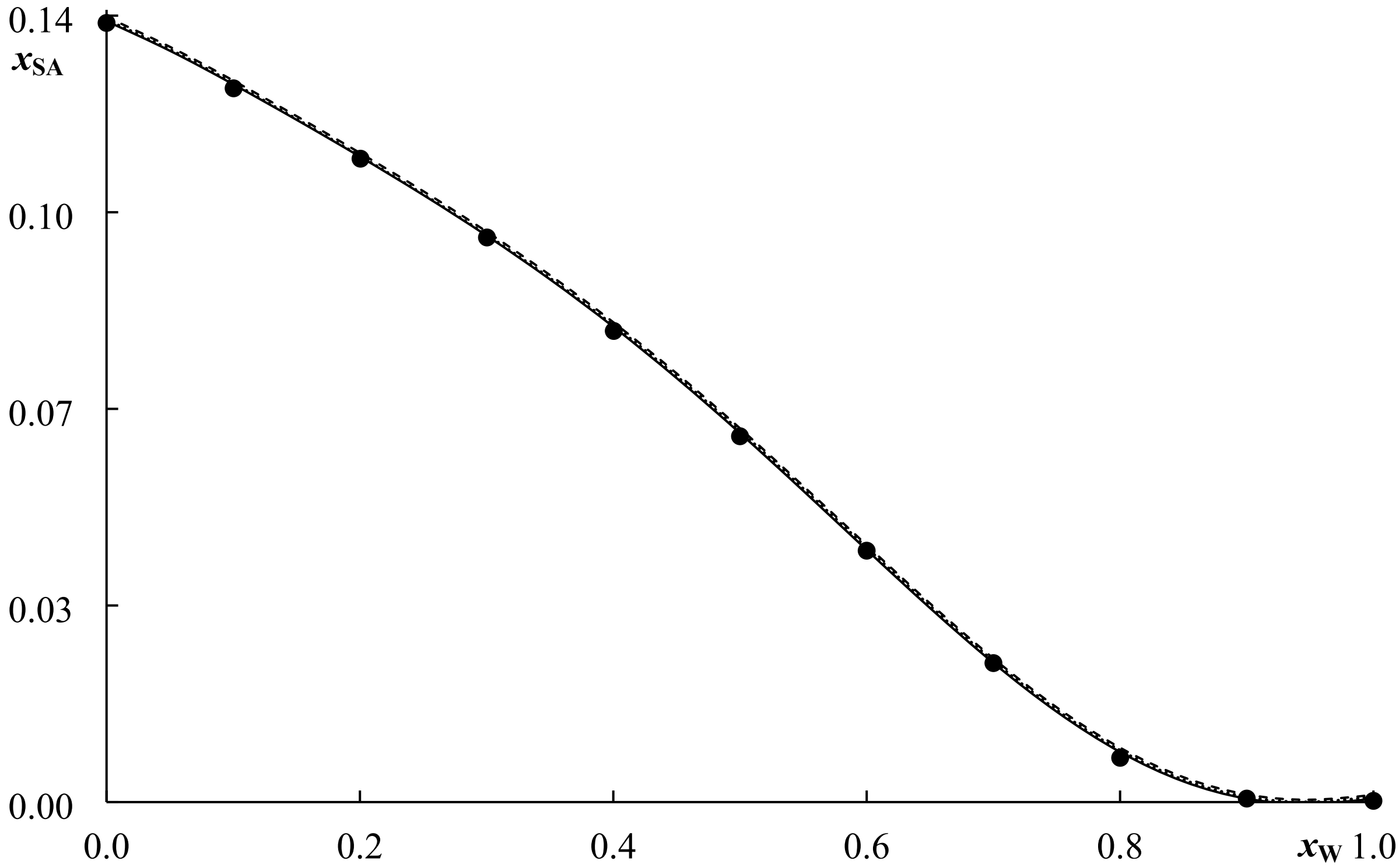

Figure S7. Solubility of salicylic acid (x_{SA}) as a function of water mole fraction (x_W) in the solutions of water + ethanol at 293.2 K: ● experimental data; PC-SAFT is the continuous line; ASOG is the dashed line; PSRK is the doted line

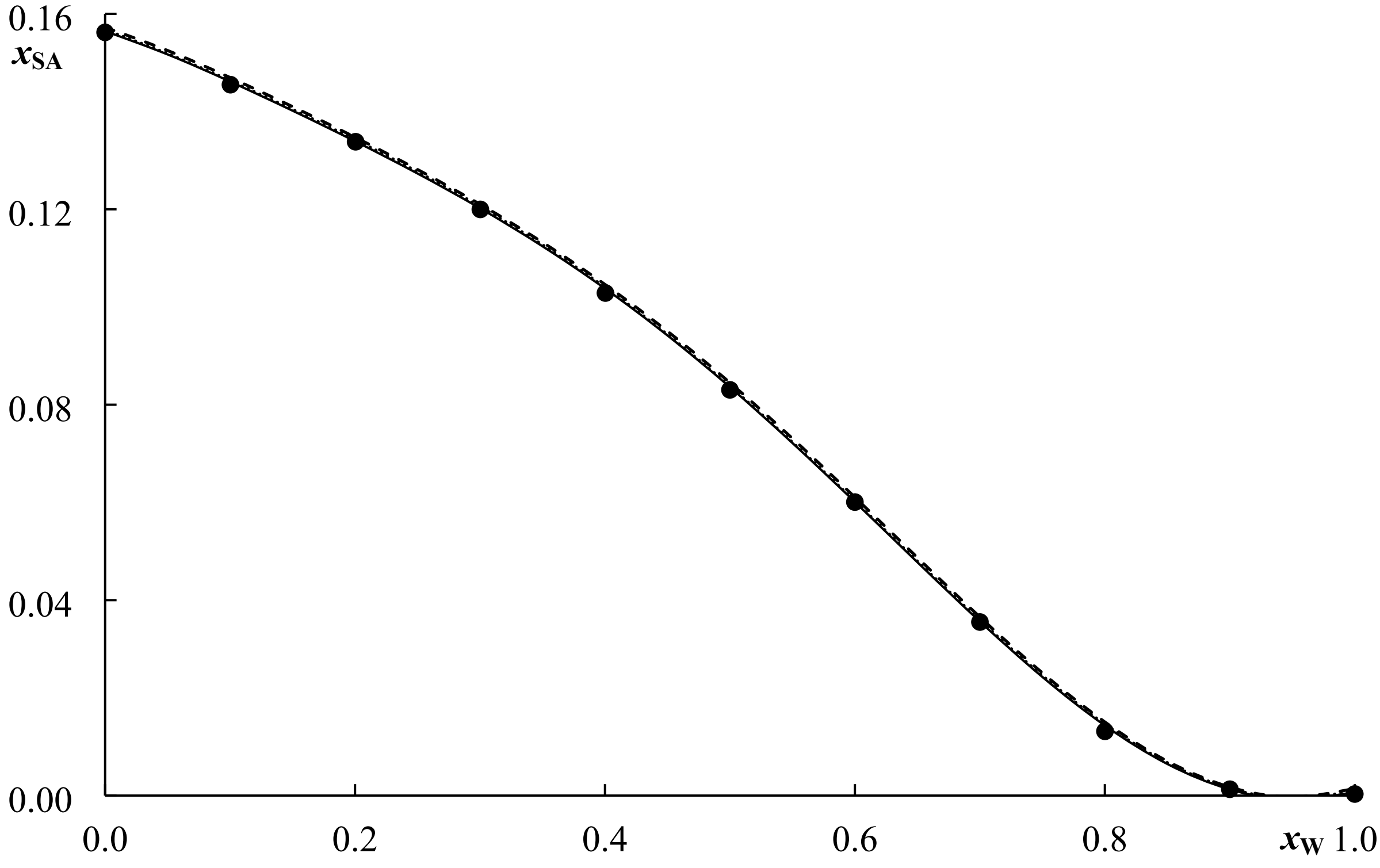

Figure S8. Solubility of salicylic acid (x_{SA}) as a function of water mole fraction (x_W) in the solutions of water + ethanol at 303.2 K: ● experimental data; PC-SAFT is the continuous line; ASOG is the dashed line; PSRK is the doted line

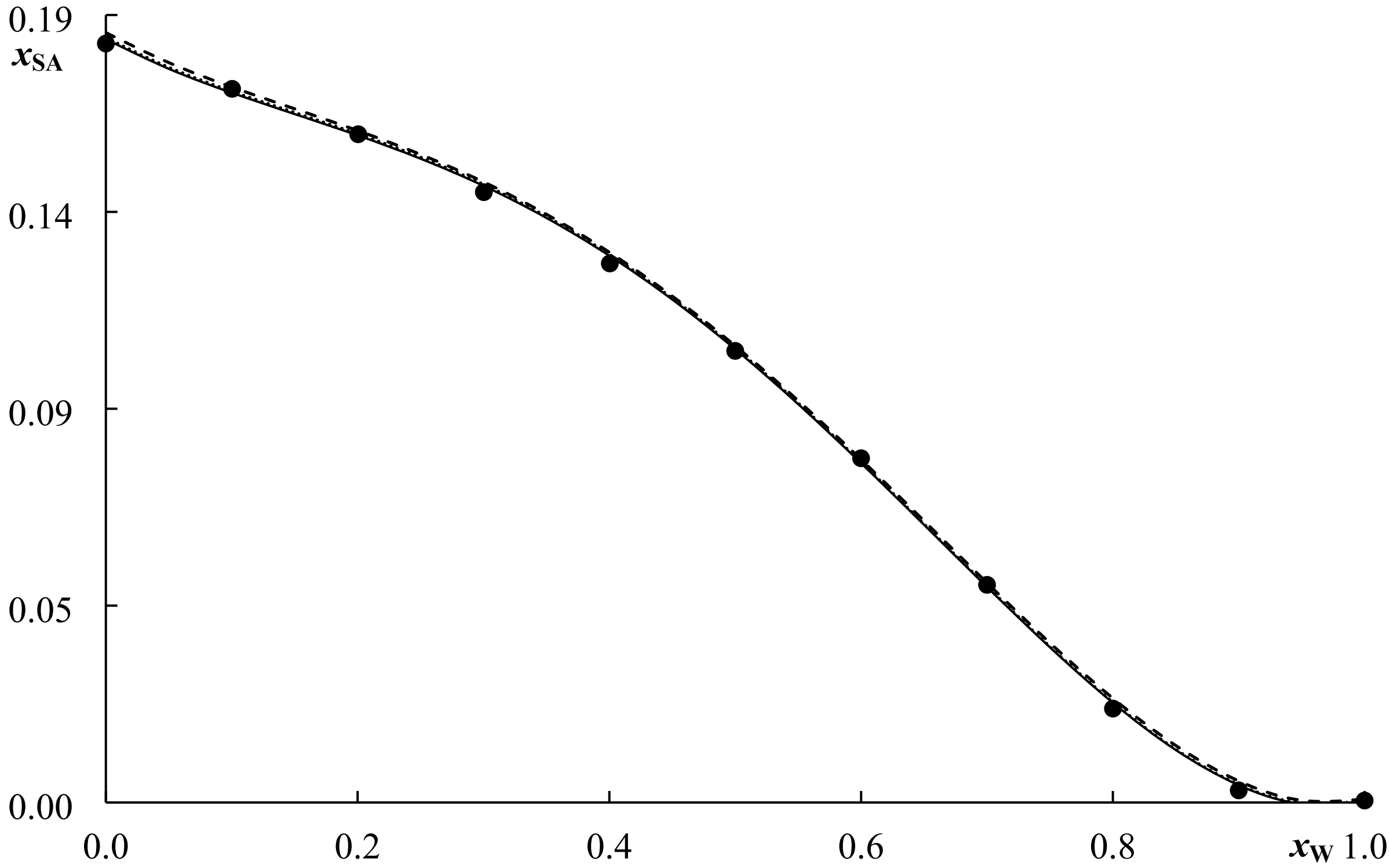

Figure S9. Solubility of salicylic acid (x_{SA}) as a function of water mole fraction (x_W) in the solutions of water + ethanol at 313.2 K: ● experimental data; PC-SAFT is the continuous line; ASOG is the dashed line; PSRK is the doted line

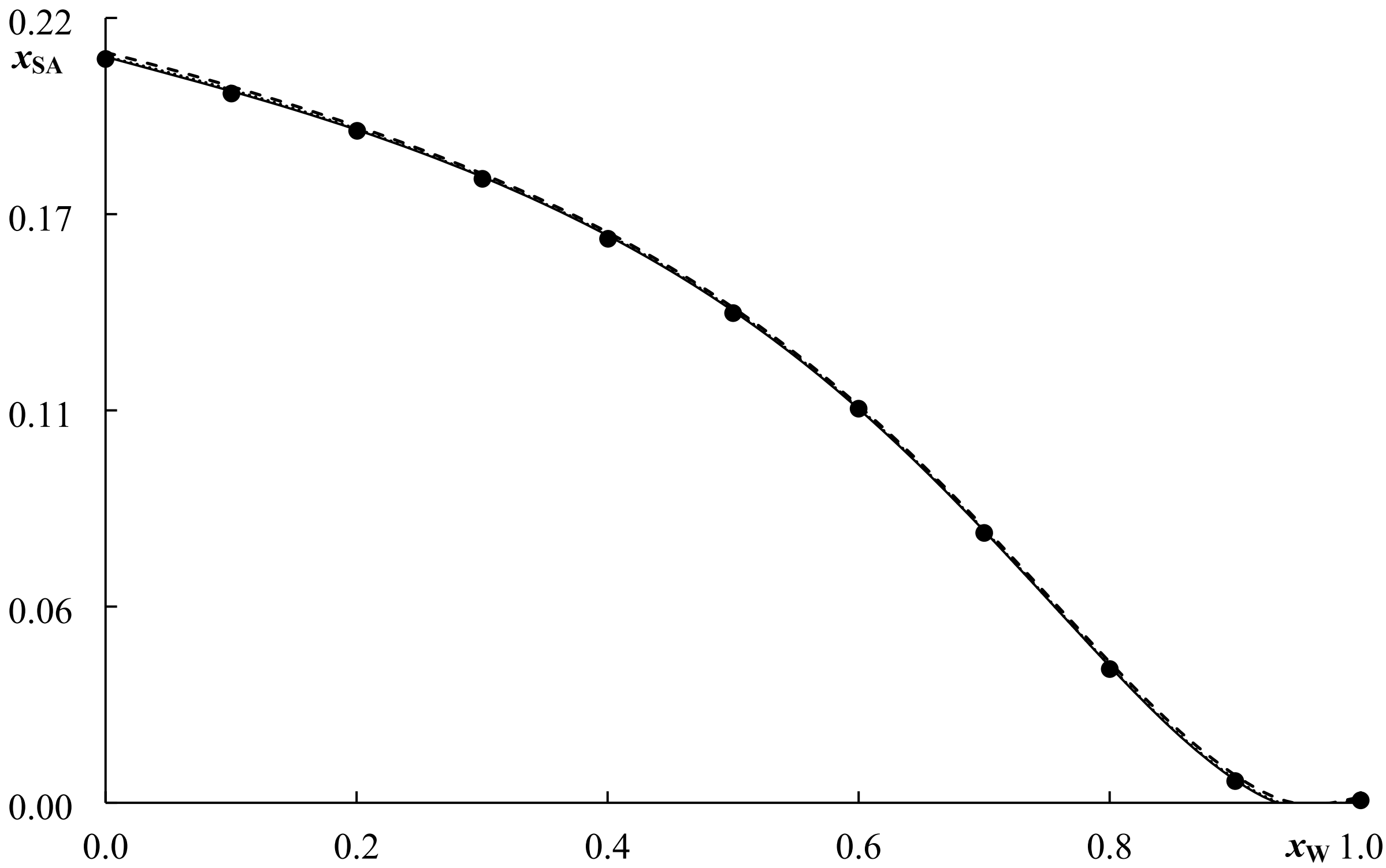

Figure S10. Solubility of salicylic acid (x_{SA}) as a function of water mole fraction (x_W) in the solutions of water + ethanol at 323.2 K: ● experimental data; PC-SAFT is the continuous line; ASOG is the dashed line; PSRK is the doted line

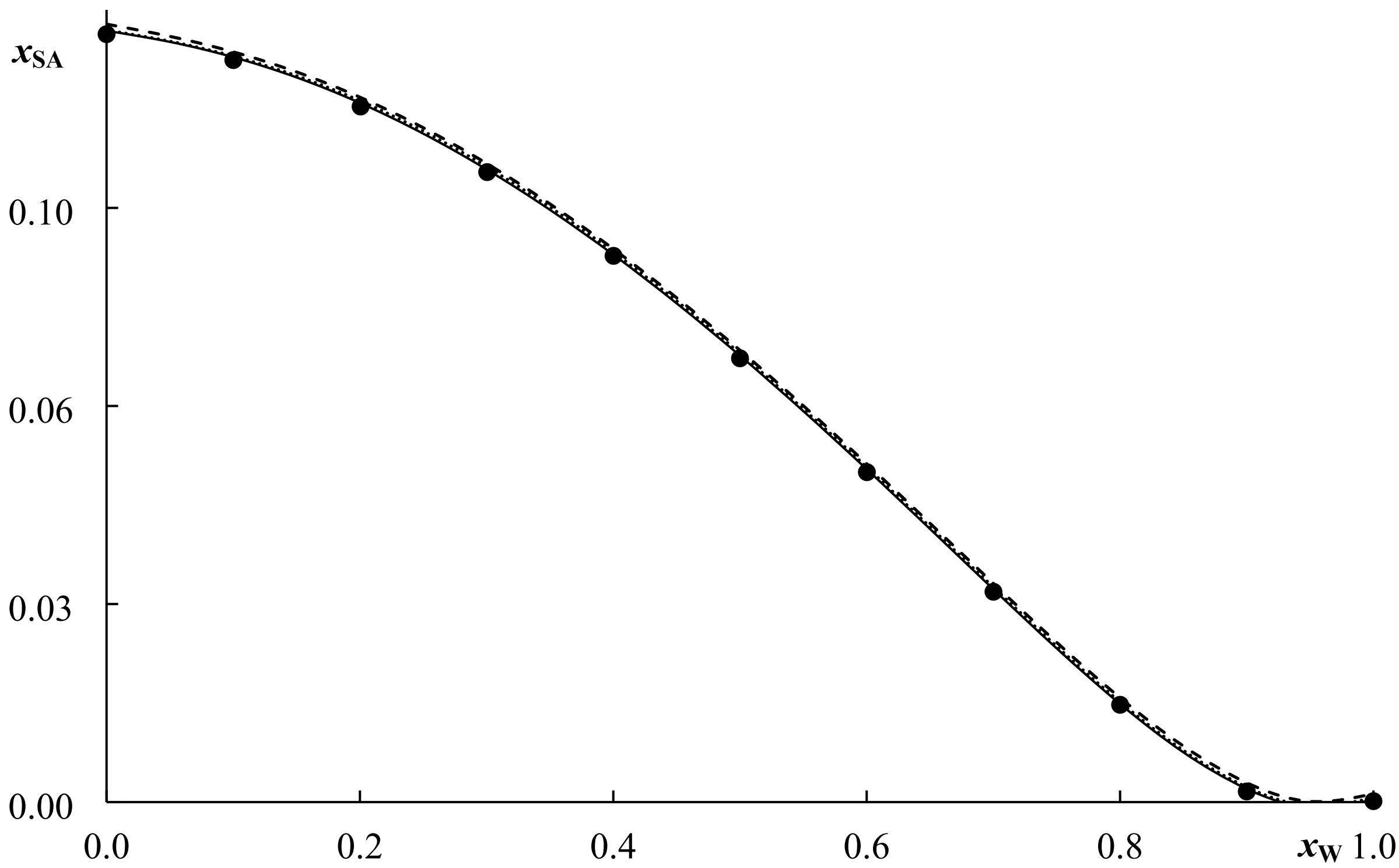

Figure S11. Solubility of salicylic acid (x_{SA}) as a function of water mole fraction (x_W) in the solutions of water + 2-propanol at 283.2 K: ● experimental data; PC-SAFT is the continuous line; ASOG is the dashed line; PSRK is the doted line

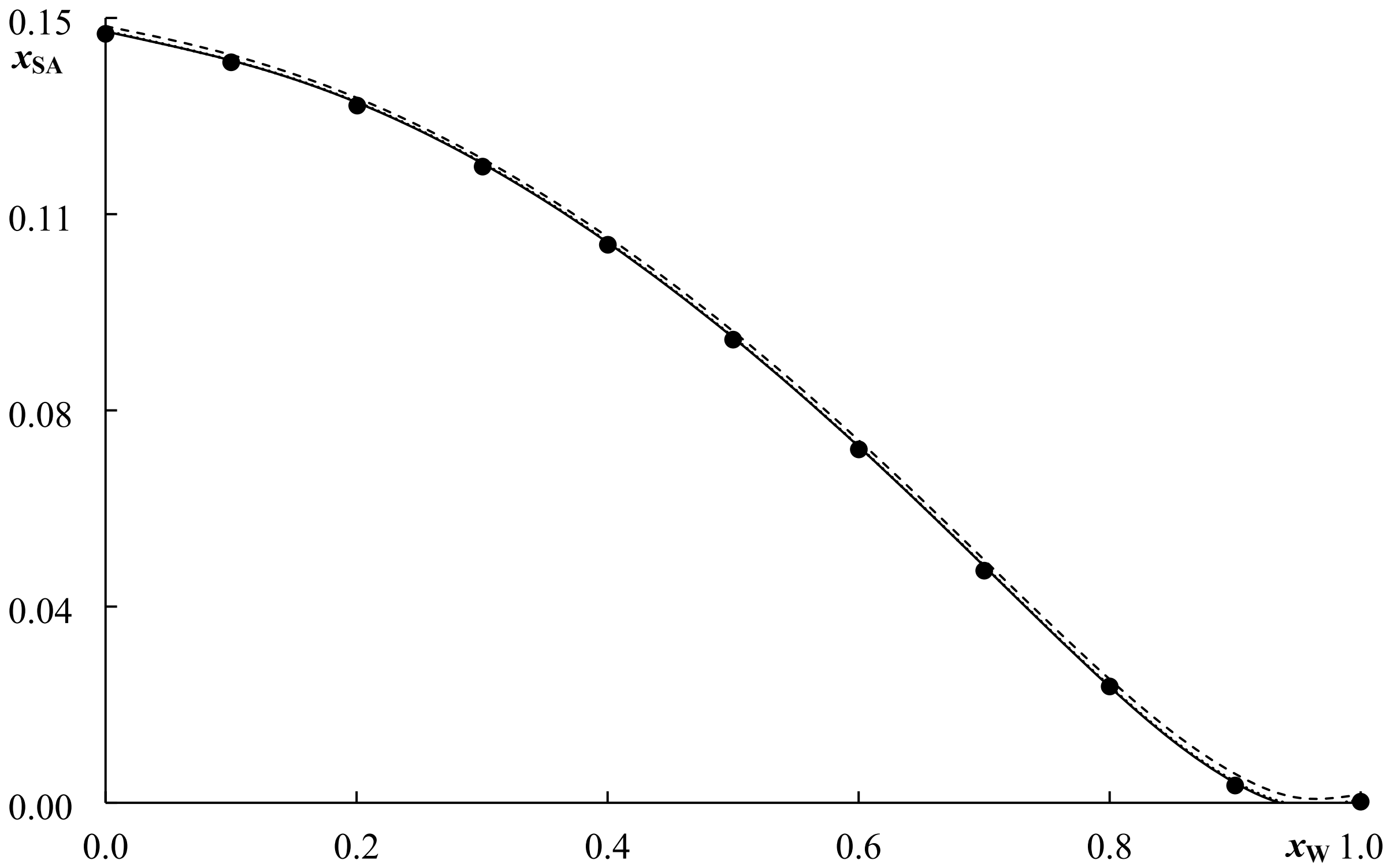

Figure S12. Solubility of salicylic acid (x_{SA}) as a function of water mole fraction (x_W) in the solutions of water + 2-propanol at 293.2 K: ● experimental data; PC-SAFT is the continuous line; ASOG is the dashed line; PSRK is the doted line

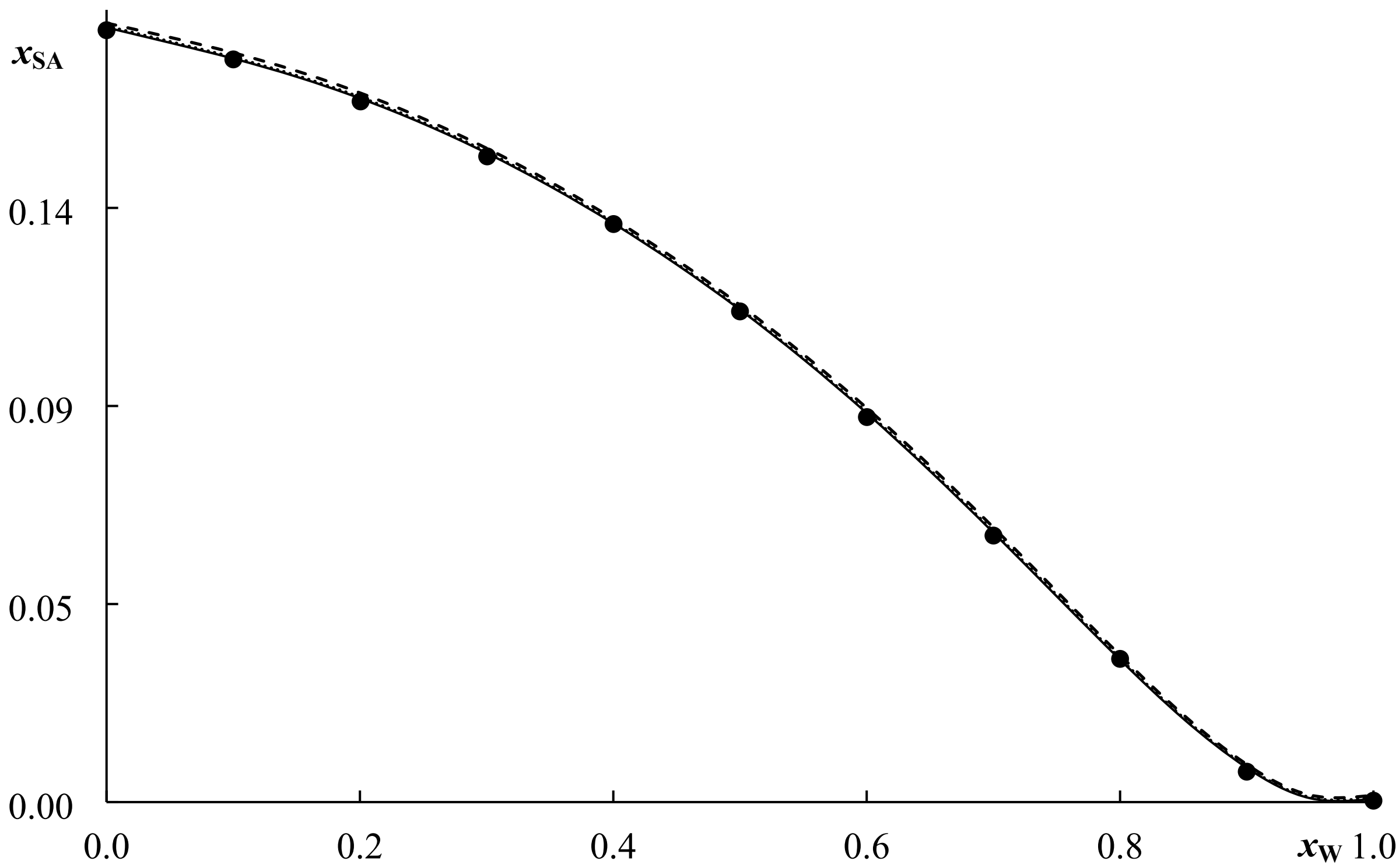

Figure S13. Solubility of salicylic acid (x_{SA}) as a function of water mole fraction (x_W) in the solutions of water + 2-propanol at 303.2 K: ● experimental data; PC-SAFT is the continuous line; ASOG is the dashed line; PSRK is the doted line

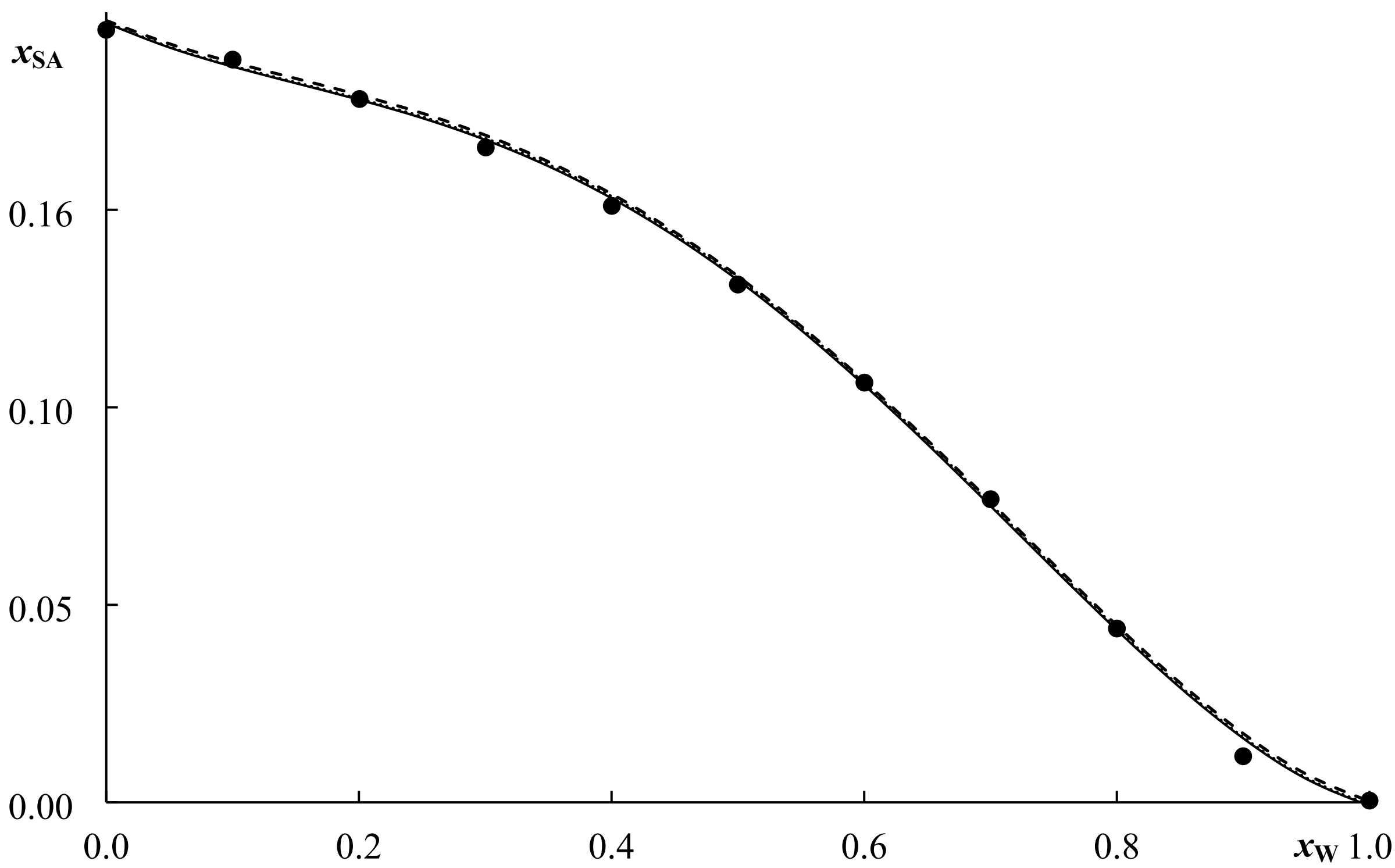

Figure S14. Solubility of salicylic acid (x_{SA}) as a function of water mole fraction (x_W) in the solutions of water + 2-propanol at 313.2 K: ● experimental data; PC-SAFT is the continuous line; ASOG is the dashed line; PSRK is the doted line

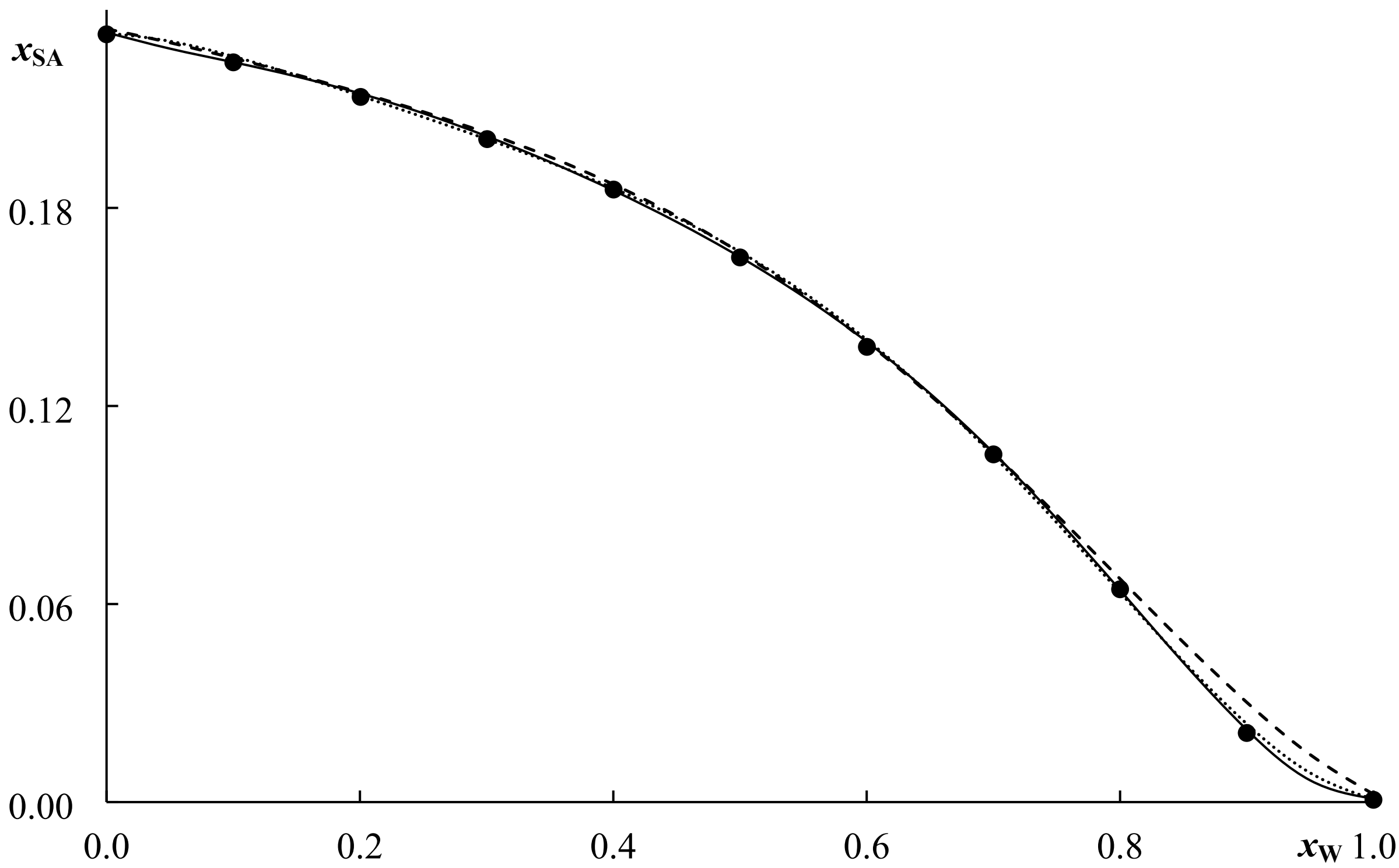

Figure S15. Solubility of salicylic acid (x_{SA}) as a function of water mole fraction (x_W) in the solutions of water + 2-propanol at 323.2 K: ● experimental data; PC-SAFT is the continuous line; ASOG is the dashed line; PSRK is the doted line

REFERENCES

(1) DIPPR Information and Data Evaluation Manager. 2000.

(2) Horstmann, S.; Jabłoniec, A.; Krafczyk, J.; Fischer, K.; Gmehling, J. PSRK Group Contribution Equation of State: Comprehensive Revision and Extension IV, Including Critical Constants and α-Function Parameters for 1000 Components. *Fluid Phase Equilib.* **2005**, *227* (2), 157–164. https://doi.org/10.1016/j.fluid.2004.11.002.

(3) Nannoolal, Y.; Rarey, J.; Ramjugernath, D. Estimation of Pure Component Properties. *Fluid Phase Equilib.* **2007**, *252* (1–2), 1–27. https://doi.org/10.1016/j.fluid.2006.11.014.

(4) Tahami, S.; Ghasemitabar, H.; Movagharnejad, K. Estimation of the Acentric Factor of Organic Compounds via a New Group Contribution Method. *Fluid Phase Equilib.* **2019**, *499*, 112246. https://doi.org/10.1016/j.fluid.2019.112246.

(5) Tochigi, K.; Tiegs, D.; Gmehling, J.; Kojima, K. Determination of New ASOG Parameters. *J. Chem. Eng. Jpn.* **1990**, *23* (4), 453–463. https://doi.org/10.1252/jcej.23.453.
